# ACSL4‐Dependent Lysosomal Lipid Peroxidation Links WTAP‐Mediated m^6^A Modification to Intervertebral Disc Degeneration

**DOI:** 10.1002/advs.77401

**Published:** 2026-08-29

**Authors:** Shu Jia, Xu Gao, Laimin Zhu, Haixia Song, Jing Wu, Shang Chen, Sheng Gao, Lu Zhang, Chunyang Meng

**Affiliations:** ^1^ Clinical Research Team of Spine & Spinal Cord Diseases Clinical Medical Research Center Affiliated Hospital of Jining Medical University Jining Medical University Jining Shandong China; ^2^ Department of Spine Surgery Affiliated Hospital of Jining Medical University Jining Medical University Jining Shandong China; ^3^ Department of Medical Imaging Affiliated Hospital of Jining Medical University Jining Medical University Jining Shandong China; ^4^ Department of Thoracic Surgery Affiliated Hospital of Jining Medical University Jining Medical University Jining Shandong China

**Keywords:** ACSL4, intervertebral disc degeneration, lipid peroxidation, lysosomal membrane permeabilization, N6‐methyladenosine

## Abstract

Intervertebral disc degeneration (IVDD) constitutes a leading contributor to the development of low back pain, yet its epigenetic regulation remains unclear. Here, we identify a mechanism by which N6‐methyladenosine (m^6^A) RNA modification contributes to lysosomal membrane permeabilization (LMP) in IVDD. Single‐cell transcriptomic analysis of human nucleus pulposus (NP) tissues shows an enrichment of lysosomal pathway genes in severely degenerated NP cells, along with LMP and disrupted mitophagy flux. We further show that interleukin‐1β (IL‐1β) induces LMP, disrupts mitophagic flux, and causes mitochondrial dysfunction in NP cells. IL‐1β enhances WTAP, a key part of the m^6^A methyltransferase complex, essential for m^6^A methylation of ACSL4 mRNA. The m^6^A reader IGF2BP2 then binds and stabilizes the modified transcripts, leading to increased ACSL4 expression. Consequently, lysosomal lipid peroxidation is promoted through accumulation of arachidonic acid‐containing phospholipids, resulting in LMP. In a rat model, intradiscal delivery of AAV9‐shWTAP attenuates disc degeneration, with preserved disc height, improved extracellular matrix synthesis, and restored lysosomal function and mitophagy. Our study reveals the WTAP/ACSL4/IGF2BP2 axis as a mediator of IL‐1β‐induced LMP via m^6^A‐dependent lipid peroxidation, highlighting a potential therapeutic target for IVDD.

## Graphical abstract

IL‐1β upregulates the protein level of WTAP, which promotes the m6A modification of ACSL4 mRNA in an IGF2BP2‐dependent manner, thereby enhancing its stability. The increased ACSL4 drives lipid peroxidation, leading to lysosomal membrane permeabilization (LMP) and impaired mitophagy, which collectively accelerate intervertebral disc degeneration (IVDD). Notably, therapeutic targeting of either WTAP or ACSL4 effectively rescues LMP and mitigates IVDD progression, highlighting a promising intervention strategy.

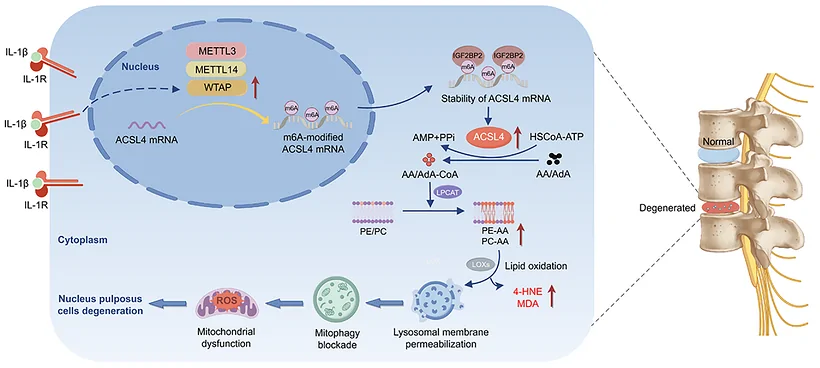

## 1 Introduction

Low back pain, a common and frequent disease, seriously affects the quality of life of approximately 632 million people worldwide, and brings a heavy economic burden to families and society [1, 2, 3, 4]. A major etiological factor in low back pain is lumbar disc herniation, a condition commonly arising from intervertebral disc degeneration (IVDD) [5, 6]. During degeneration, the inner nucleus pulposus (NP) experiences a gradual reduction in water and proteoglycans, altering the structural and mechanical properties of the intervertebral disc [7]. IVDD often leads to annulus fibrosus (AF) rupture, NP herniation, and symptoms such as nerve root compression and discogenic pain. While the pathogenesis of IVDD includes trauma, senescence, and excessive loading, the underlying molecular mechanism of IVDD is complex and worthy of further investigation and elucidation.

Lysosomes, containing a variety of acidic hydrolases, are responsible for the degradation of various biological macromolecules such as proteins, nucleic acids, and polysaccharides [8]. The integrity of lysosomal membranes is crucial for their function and homeostasis [9]. Lysosomal membrane permeabilization (LMP) can be induced by various stressors, including reactive oxygen radicals and photodamage, causing the release of cathepsin‐based hydrolases into the cytoplasm and leading to lysosome‐dependent cell death [10, 11, 12]. Our prior research demonstrated that oxidative stress‐induced LMP directly contributes to the pathology of human IVDD by impairing lysosomal autophagy, thereby accelerating NP cell senescence [13]. In addition, overloading could also lead to LMP, with impaired autophagic flux and death of NP cells [14]. The precise role and mechanism of LMP in IVDD pathogenesis require further investigation.

N6‐methyladenosine (m^6^A) is a conserved RNA modification occurring post‐transcriptionally in eukaryotic cells [15]. The m^6^A modification influences mRNA splicing, translation, stability, and transport through the coordinated actions of writer, eraser, and reader proteins [16]. The methyltransferase complex, comprising methyltransferase‐like 3 (METTL3), Wilms’ tumor 1‐associating protein (WTAP), and METTL14, is responsible for the installation of m^6^A marks. METTL3 acts as the catalytic core, with METTL14 and WTAP providing the RNA‐binding platform in the complex [17]. m^6^A can also be demethylated by the AlkB homolog 5 (ALKBH5) and fat mass and obesity‐associated gene (FTO) [18, 19]. Readers, including insulin‐like growth factor 2 mRNA‐binding proteins (IGF2BPs) and YT521‐B homology domain family (YTHDF) can identify m^6^A‐modified mRNAs and perform regulatory roles [20, 21]. YTHDF2 would promote m^6^A‐methylated RNAs for degradation, while IGF2BPs could stabilize the mRNAs and promote translation efficiency. A recent study investigated the m^6^A methylation pattern in NP tissues using a mouse model of intervertebral disc degeneration [22]. Yang et al. reported that WTAP‐regulated m^6^A modification of lncRNA NORAD promotes NP cell senescence and IVDD development [23]. However, whether the m^6^A epigenetic modification participates in the LMP process of NP cells and IVDD progression remains unknown.

In this study, we identified interleukin‐1β (IL‐1β)‐induced LMP damage as a potential driver of IVDD. IL‐1β stimulation heightened lysosomal membrane permeability in degenerated NP cells, disrupting mitophagy and worsening mitochondrial dysfunction. Our study identified WTAP as a key mediator of LMP via m^6^A modification on acyl‐CoA synthetase long chain family 4 (ACSL4) mRNA. IGF2BP2 recognized and increased the stability of m^6^A‐modified ACSL4 mRNA, resulting in the elevation of ACSL4 protein levels. Furthermore, upregulated ACSL4 promoted lysosomal lipid peroxidation and triggered LMP in degenerated NP cells. These findings clarify the molecular mechanism of IL‐1β‐induced LMP and highlight the WTAP/ACSL4/IGF2BP2 axis as a tractable target for IVDD therapy.

## 2 Results

### 2.1 Lysosomal Membrane Permeabilization Disrupts Mitophagy in Degenerated Intervertebral Disc

To dissect the molecular and cellular characteristics of NP cells during IVDD, we analyzed our previously published single‐cell transcriptomic dataset (GSE251686) of human NP tissues with different Pfirrmann grades. The cellular heterogeneity of NP tissues was classified by representative gene markers, of which the NP cluster was identified by SOX9, ACAN, COL2A1, KRT18, and FOXF1 (Figure 1A,B). As the degeneration degree increases, the proportion of NP cluster decreases from 65% to 39% (Figure 1C). Differentially expressed genes associated with lysosomes, including cathepsin B (CTSB), CTSD, CTSK, and CTSL, were identified in severely degenerated NP cells. KEGG pathway analysis indicated a significant enrichment in the lysosome pathway (Figure 1D,E). To confirm these findings, we analyzed an independent single‐cell dataset (GSE153066, 8 control and 8 IVDD patients) of human NP tissues, which similarly revealed a reduced NP cell proportion and consistent enrichment of the lysosome pathway in degenerated NP cells (Figure S1A–H). Compared with the mild degeneration group, the protein levels of mature CTSB and CTSD were significantly decreased in severe degeneration samples (Figure 1F). In addition, a significant reduction in the colocalization of cathepsins (CTSB and CTSD) with lysosomal associated membrane protein 1 (LAMP1) was observed in NP tissue with severe degeneration (Figure 1G). Defects in cathepsin maturation may result from mechanisms such as impaired lysosomal acidification, abnormal proton pump activity, and LMP damage. Using transmission electron microscopy (TEM), we evaluated lysosomal ultrastructural changes in NP cells exhibiting varying degeneration levels. TEM analysis revealed that severely degenerated NP cells exhibited lysosomal membrane rupture, leading to the leakage of internal substances (Figure 1H). Normal lysosomal function and quality control are essential for autophagy. Mitophagy, a type of selective autophagy, degrades damaged mitochondria and facilitates their recycling, regulating the formation of new mitochondria to sustain mitochondrial dynamics. A marked increase in mitophagy‐related proteins, including microtubule‐associated protein 1 light chain 3 (LC3)‐II/I ratio, PTEN‐induced kinase 1 (PINK1), sequestosome 1 (SQSTM1/p62), and BCL2 interacting protein 3 (BNIP3), was observed in severely degenerated NP tissues, suggesting a potential impairment of mitophagic degradation (Figure 1I). We employed L‐leucyl‐L‐leucine methyl ester hydrobromide (LLOME) to rapidly and reversibly induce LMP in order to evaluate its impact on lysosomal dysfunction and mitophagy. Cells treated with LLOME displayed a robust reduction in the colocalization percentage of CTSB/CTSD with LAMP1 compared with the control group (Figure 1J). Besides, the mature CTSB/CTSD, LC3‐II/I ratio, p62, and BNIP3 protein abundance were significantly elevated with the extension of LLOME treatment (Figure 1K). Overall, these data demonstrated the presence of LMP in severely degenerated NP tissues and subsequent impairment of mitophagic degradation.

**FIGURE 1:**
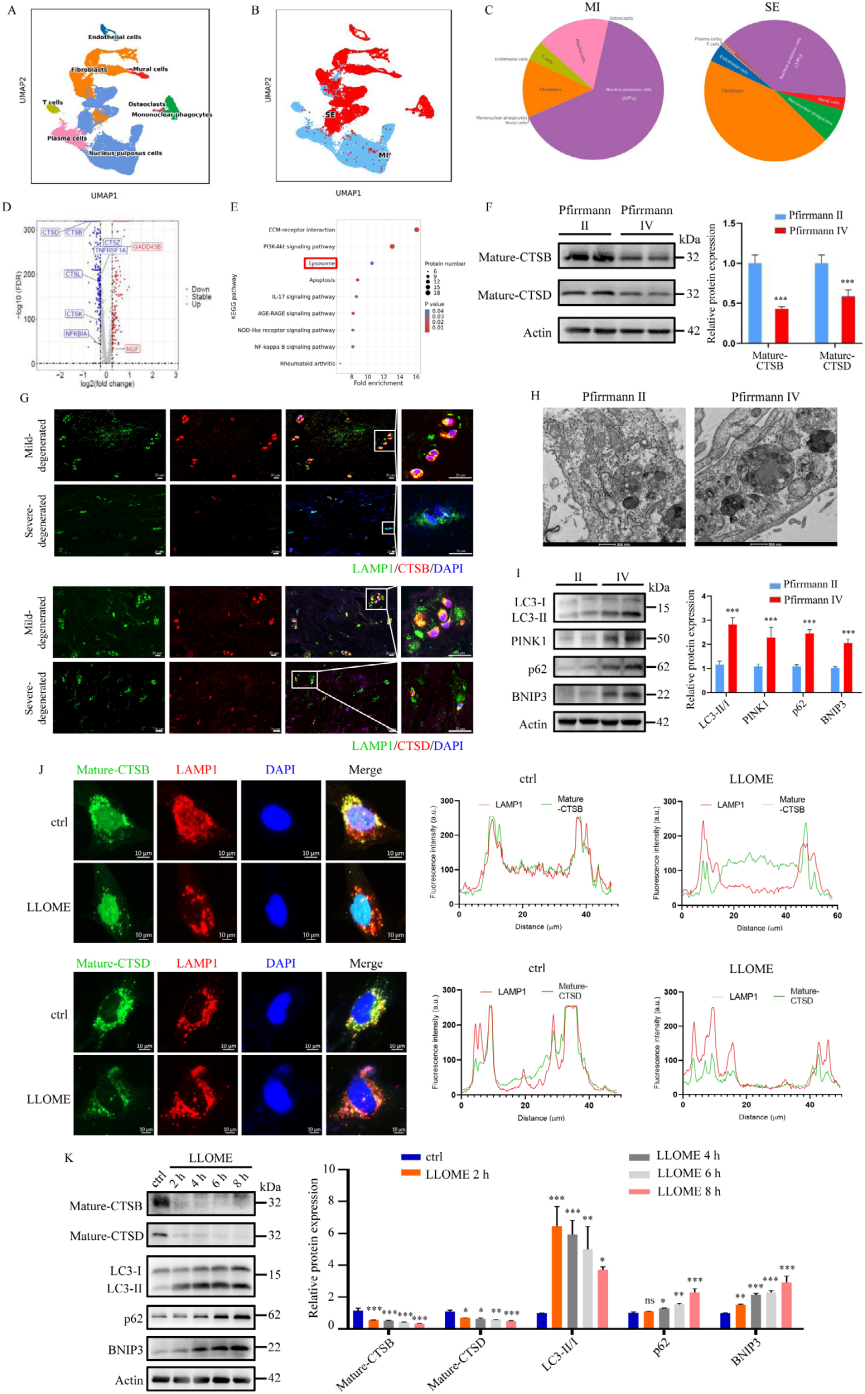
Lysosomal membrane permeabilization disrupts mitophagy in degenerated intervertebral disc. (A) UMAP visualization of human nucleus pulposus (NP) tissues revealed eight distinct clusters. Each dot represents a distinct colored cell cluster. (B) UMAP visualization illustrating the cellular makeup of NP tissues across various degenerative grades. MI indicates mild degeneration, while SE denotes severe degeneration. (C) Pie chart displaying the composition of cell clusters in MI and SE NP tissues. (D) Volcano plot illustrating differentially expressed genes (DEGs) in NP cells comparing MI and SE groups. (E) Dot plot presenting the enriched KEGG pathways of DEGs in NP clusters. (F) Mature‐CTSB and CTSD protein levels in Pfirrmann II and IV NP tissues, two‐tailed unpaired *t*‐test. (G) Typical IF images of LAMP1 co‐stained with CTSB and CTSD in mild‐ and severe‐degenerated NP tissues. (Scale bar = 20 µm) (H) Typical TEM images of Pfirrmann II and IV NP tissues. (Scale bar = 500 nm) (I) Western blotting of mitophagy markers LC3‐I, LC3‐II, PINK1, p62, and BNIP3 in Pfirrmann II and IV NP tissues, two‐tailed unpaired *t*‐test. (J) Typical IF images of LAMP1 co‐stained with mature‐CTSB and CTSD in NP cells treated with LLOME (1 mM, 8 h). (Scale bar = 10 µm) (K) Western blotting of mature‐CTSB, mature‐CTSD, LC3‐I, LC3‐II, p62, and BNIP3 in NP cells exposed to LLOME for 0, 2, 4, 6, and 8 h, one‐way ANOVA with Tukey‐Kramer test. All values are expressed as mean ± SD. The error bars correspond to the means from three independent replicates. ^*^ *p* < 0.05, ^**^ *p* < 0.01 and ^***^ *p* < 0.001.

### 2.2 IL‐1β Induces LMP and Impairs Mitophagy Degradation in NP Cells

Within the inflammatory microenvironment of degenerated discs, NP cells undergo aging, apoptosis, and autophagy. Considering the role of interleukin‐1beta (IL‐1β), we evaluated its effects on LMP and mitophagy in NP cells. Exposure to IL‐1β resulted in a time‐dependent reduction in collagen II expression and a marked upregulation of MMP13 (Figure 2A). IL‐1β treatment provoked lysosomal membrane rupture, resulting in the leakage of internal substances (Figure 2B). In human NP tissues of different Pfirrmann grades, immunofluorescence co‐staining showed that IL‐1β levels positively correlated with galectin‐3‐defined LMP severity and IVDD pathological grade (Figure S2A). Following LMP, galectin‐3/LGALS3 binds to exposed intraluminal galactose‐containing glycans, forming discernible puncta on damaged lysosomes. Lysosomal membrane perforation was analyzed by visualizing the formation of LGALS3 puncta. We observed that IL‐1β treatment resulted in the accumulation of approximately sixty LGALS3 puncta co‐localized with LAMP1 per cell, in contrast to almost none in control cells (Figure 2C). Prolonged exposure to IL‐1β significantly decreased the protein levels of mature CTSB and CTSD (Figure 2D). Concurrently, the expression of mitophagy‐related proteins, including PINK1, p62, BNIP3, and the LC3‐II/I ratio, was remarkably up‐regulated, a finding corroborated by immunofluorescence assays (Figure 2E–G). To determine whether this accumulation reflects enhanced autophagosome synthesis or impaired degradative flux, NP cells were treated with bafilomycin A1 (BafA1), an inhibitor of vacuolar H^+^‐ATPase that blocks autophagosome‐lysosome fusion and lysosomal degradation. In control cells, both IL‐1β and BafA1 treatment separately led to a marked accumulation of LC3‐II/I, p62, and BNIP3 (Figure 2H). However, in IL‐1β‐treated cells, co‐incubation with BafA1 yielded protein levels comparable to BafA1 alone (Figure 2H). To directly monitor autophagic flux, we employed a mCherry‐GFP‐LC3 tandem reporter assay. Both IL‐1β and BafA1 treatment individually increased the number of LC3‐positive yellow puncta, whereas co‐treatment with IL‐1β and BafA1 did not further increase yellow puncta compared to BafA1 alone (Figure 2I). To confirm the specificity of IL‐1β‐induced defects in lysosomal and mitophagic degradation, NP cells were co‐treated with Anakinra, an IL‐1 receptor antagonist. Anakinra treatment largely reversed the IL‐1β‐induced increase in LC3‐positive yellow puncta (Figure S2B). Anakinra consistently inhibited the IL‐1β‐induced decrease in mature CTSB/CTSD, and significantly reduced the increase in p62, BNIP3, and LC3‐II/I ratio (Figure S2C). These results confirm that the observed LMP and mitophagic degradation impairment are specifically driven by IL‐1 receptor activation. IL‐1β‐treated NP cells exhibited a sharp decline in mitochondrial membrane potential, as indicated by Rhodamine 123 staining, aligning with impaired mitophagy (Figure 2J) and a significant increase in mitochondrial ROS levels as detected by MitoSOX Red (Figure 2K). Collectively, these data demonstrated that IL‐1β induces LMP and disrupts mitophagic flux, resulting in the accumulation of dysfunctional mitochondria in NP cells.

**FIGURE 2:**
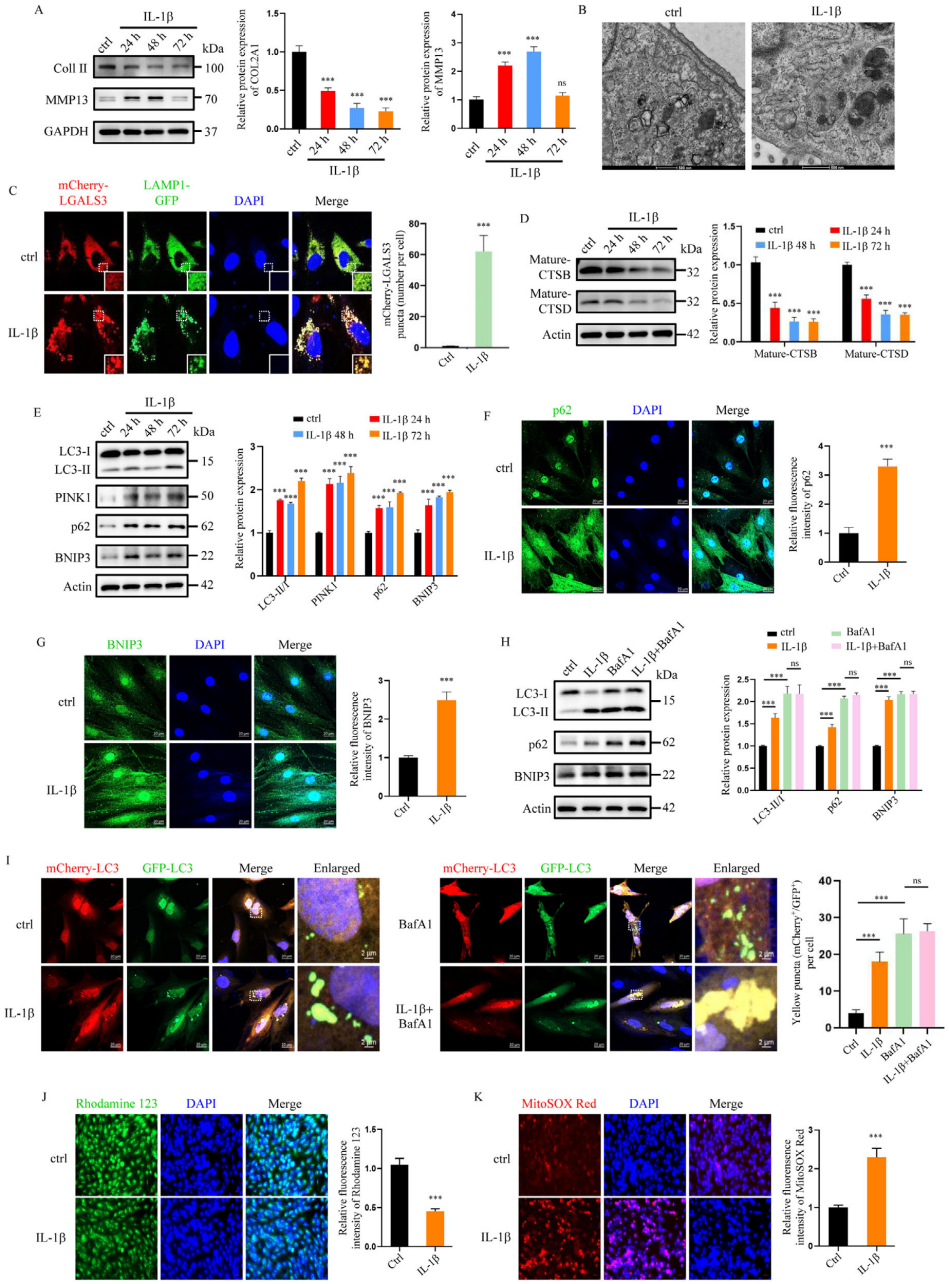
IL‐1β induces LMP and impairs mitophagy degradation in NP cells. (A) Western blotting of collagen II and MMP13 under 20 ng/mL IL‐1β stimulation for 0, 24, 48, and 72 h, one‐way ANOVA with Tukey‐Kramer test. (B) Typical TEM images depicting NP cells after 72‐h stimulation with IL‐1β treatment or under control conditions. (Scale bar = 500 nm) (C) Typical confocal microscopy images of NP cells transfected with mCherry‐LGALS3 and LAMP1‐GFP plasmids followed by IL‐1β treatment, two‐tailed unpaired *t*‐test. (Scale bar = 10 µm) (D) Mature‐CTSB and CTSD protein levels in NP cells under the corresponding IL‐1β treatment, one‐way ANOVA with Tukey‐Kramer test. (E) Western blotting of mitophagy markers LC3‐I, LC3‐II, PINK1, p62, and BNIP3 in NP cells under the corresponding IL‐1β treatment, one‐way ANOVA with Tukey‐Kramer test. (F, G) Typical IF images of p62 and BNIP3 in NP cells with or without IL‐1β treatment, two‐tailed unpaired *t*‐test. (Scale bar = 20 µm) (H) Western blotting of LC3‐I, LC3‐II, p62, and BNIP3 in NP cells with or without the presence of IL‐1β (20 ng/mL, 72 h) and BafA1 (100 nm, 4 h), two‐way ANOVA with Tukey‐Kramer test. (I) Autophagic flux of the NP cells treated as described in (H) was evaluated via the mCherry‐GFP‐LC3 reporter assay. Yellow puncta (mCherry^+^/GFP^+^) indicate autophagosomes and impaired autolysosome degradation capacity; two‐way ANOVA with Tukey‐Kramer test. (Scale bar = 20 µm, Enlarged scale bar = 2 µm) (J) Typical IF images of Rhodamine 123 staining in NP cells with or without IL‐1β stimulation, two‐tailed unpaired *t*‐test. (Scale bar = 50 µm) (K) Typical IF images of MitoSOX Red staining (5 µm, 30 min) in NP cells with or without IL‐1β treatment, two‐tailed unpaired *t*‐test. (Scale bar = 50 µm) Values are shown as mean ± SD. ^*^ vs Ctrl, ^***^ *p* < 0.001, ns: no significant difference.

### 2.3 m^6^A Modification of ACSL4 Mediates LMP and Mitophagic Flux Impairment

Methylated RNA immunoprecipitation sequencing (MeRIP‐Seq) was conducted to study the impact of m^6^A modification on LMP in NP cells by analyzing RNAs with varying transcript levels and m^6^A peaks. A total of 41 866 m^6^A peaks were identified in control NPCs, while 39 651 peaks were found in IL‐1β‐treated NPCs (Table S1). Analysis of peak distribution revealed a predominant enrichment of m^6^A sites in exons and 3’UTRs (Figure 3A). The consensus motif of RRACU (R = A, G) was enriched in m^6^A‐purified peaks (Figure 3B). GO and KEGG analyses of differential peak genes were enriched in terms related to autophagy (Figure S3A,B). Correlation analysis showed that m^6^A modification of ACSL4 was significantly increased in IL‐1β‐treated NPCs, accompanied by elevated mRNA expression, as validated by MeRIP‐qPCR and qRT‐PCR (Figure 3C–E). Furthermore, western blot and immunofluorescence results confirmed that prolonged IL‐1β exposure also upregulated the protein level of ACSL4 (Figure 3F,G). Loss‐of‐function experiments demonstrated that ACSL4 deficiency rescued the IL‐1β‐induced downregulation of mature CTSB and CTSD protein levels, and reduced the number of LGALS3 puncta‐labeled damaged lysosomes (Figure 3H,I). Moreover, ACSL4 silencing mitigated the accumulation of LC3‐II, p62, and BNIP3 in IL‐1β‐stimulated cells (Figure 3J–M). We also observed that ACSL4 knockdown attenuated IL‐1β‐induced mitochondrial fragmentation, partially restoring the tubular mitochondrial network (Figure 3N). Collectively, these findings indicated that m^6^A‐mediated upregulation of ACSL4 contributed to LMP and the disruption of mitophagic flux in NP cells.

**FIGURE 3:**
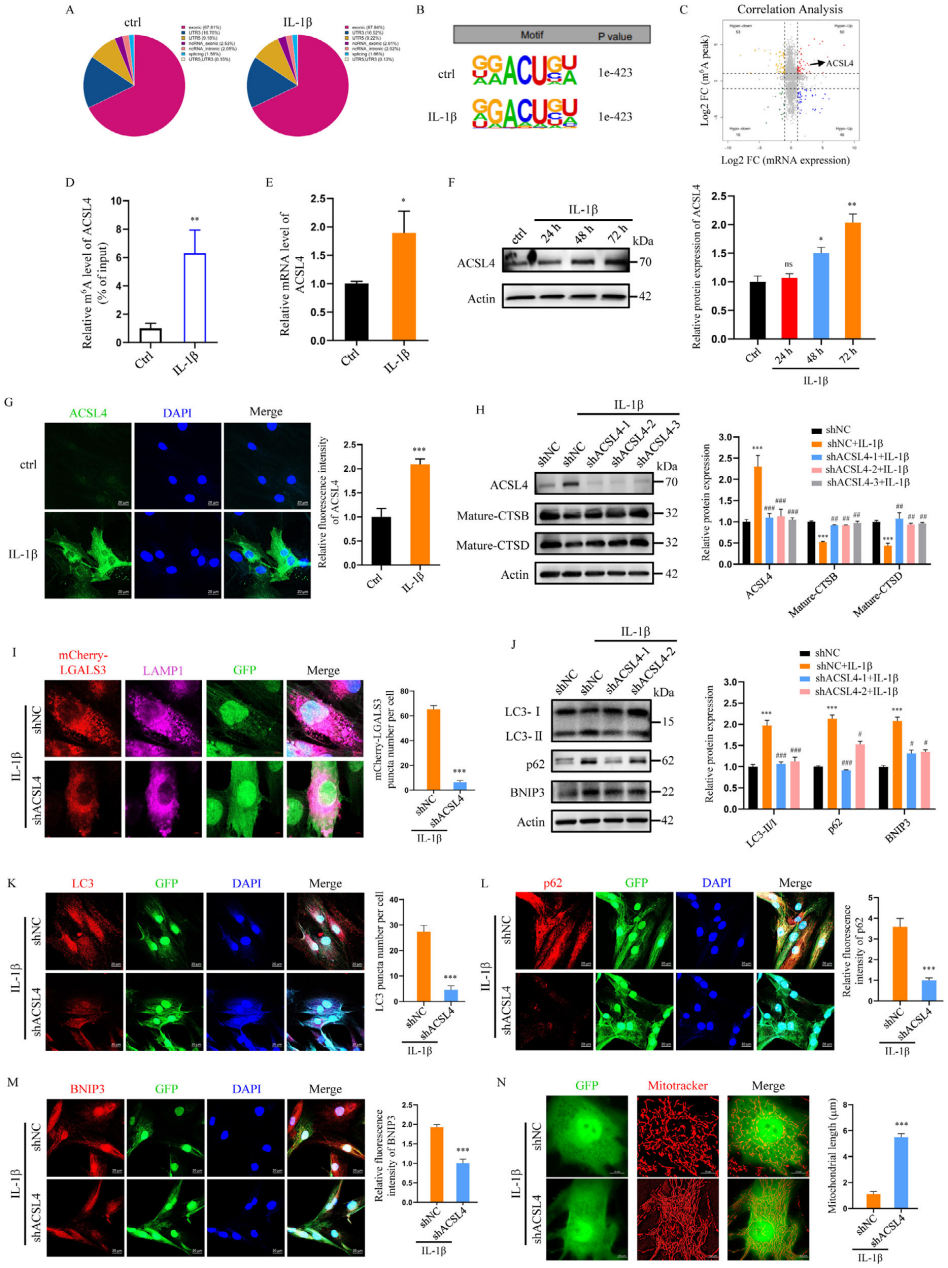
m^6^A modification of ACSL4 mediates LMP and mitophagic flux impairment. (A) m^6^A peak distribution across gene regions from MeRIP‐seq data in normal and IL‐1β‐challenged NPCs. (B) Typical motif of m^6^A‐modified transcripts in normal and IL‐1β‐challenged NPCs. (C) Correlation analysis of m^6^A peak and mRNA expression levels. (D) Relative m^6^A level of ACSL4 by MeRIP‐qPCR in NP cells with or without IL‐1β treatment, two‐tailed unpaired *t*‐test. (E) Relative level of ACSL4 mRNA by qRT‐PCR in NP cells with or without IL‐1β stimulation, two‐tailed unpaired *t*‐test. (F) Western blotting of ACSL4 protein levels in NP cells under the corresponding IL‐1β treatment, one‐way ANOVA with Tukey‐Kramer test. (G) Typical IF images of ACSL4 in NP cells with or without IL‐1β treatment, two‐tailed unpaired *t*‐test. (Scale bar = 20 µm) (H) Protein levels of ACSL4, mature‐CTSB, and mature‐CTSD in IL‐1β‐treated NP cells by western blot, with or without ACSL4 silencing, two‐way ANOVA with Tukey‐Kramer test. (I) Typical confocal microscopy images of NP cells transfected with mCherry‐LGALS3 plasmids, then infected with negative control (NC) or ACSL4 knockdown lentivirus labeled with GFP, and finally stimulated with IL‐1β for 72 h, two‐tailed unpaired *t*‐test. (Scale bar = 5 µm) (J) Western blotting of LC3‐I, LC3‐II, p62, and BNIP3 in IL‐1β‐challenged NP cells with or without ACSL4 knockdown, two‐way ANOVA with Tukey‐Kramer test. Values are expressed as mean ± SD. ^*^ vs shNC, ^***^ *p* < 0.001; # vs shNC+IL‐1β, #p < 0.05, ##p < 0.01, ###p < 0.001. (K–M) Typical IF images of LC3, p62, and BNIP3 in IL‐1β‐treated NP cells with ACSL4 silencing or not, two‐tailed unpaired *t*‐test. (Scale bar = 20 µm) (N) Super‐resolution microscopy images of Mitotracker staining (100 nm, 30 min) in IL‐1β‐treated NP cells with or without ACSL4 knockdown, two‐tailed unpaired *t*‐test. (Scale bar = 10 µm).

### 2.4 WTAP‐Mediated m^6^A Modification of ACSL4 Drives LMP and Disrupts Mitophagic Flux

To explore the mechanistic basis of m^6^A modification, we analyzed the methyltransferases and demethylases expression in NPCs. Western blot analysis demonstrated a notable increase in WTAP methyltransferase levels in IL‐1β‐stimulated NP cells, supported by immunofluorescence and aligning with the RNA‐sequencing data (Figure 4A–C). To investigate the role of WTAP in ACSL4 m^6^A modification, we silenced WTAP using three distinct lentiviral shRNAs. Knockdown of WTAP abrogated the hypermethylation of ACSL4 in NPCs treated with IL‐1β (Figure 4D). Moreover, WTAP deficiency markedly suppressed the IL‐1β‐induced increased abundance of ACSL4 at both mRNA and protein levels (Figure 4E,F). We next assessed the impact of WTAP silencing on LMP in NPCs. Depletion of WTAP led to elevated levels of mature cathepsins (CTSB and CTSD) and a reduction in LGALS3 puncta‐positive damaged lysosomes compared to IL‐1β‐treated controls (Figure 4G,H). Finally, we examined the influence of WTAP on mitophagic flux and mitochondrial morphology. WTAP silencing markedly reduced the IL‐1β‐induced increase in LC3‐II, p62, and BNIP3 levels (Figure 4G). Consistent with restored mitophagic flux, WTAP knockdown also ameliorated IL‐1β‐induced mitochondrial fragmentation, partially restoring the tubular mitochondrial network (Figure 4I). Conversely, overexpression of ACSL4 substantially reversed the protective effects of WTAP silencing, leading to re‐emergence of LGALS3 puncta (Figure 4J), decreased levels of mature cathepsin B/D proteins, and increased accumulation of LC3‐II/I, p62, and BNIP3 (Figure 4K and Figure S4). Taken together, these data confirmed that WTAP‐directed m^6^A modification of ACSL4 acted as an upstream trigger for LMP and mitophagic impairment in NPCs.

**FIGURE 4:**
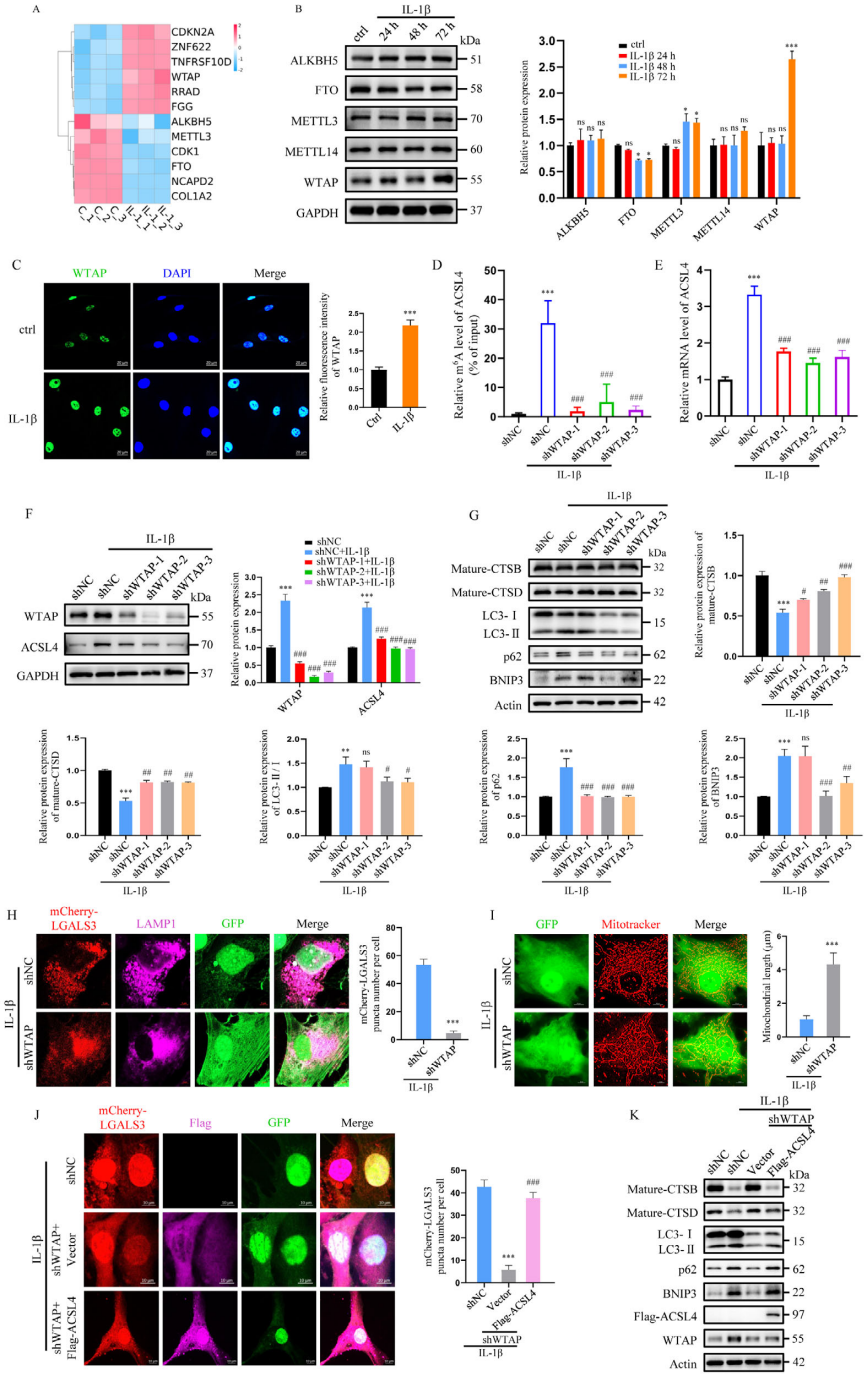
WTAP‐mediated m^6^A modification of ACSL4 drives LMP and disrupts mitophagic flux. (A) Heatmap illustrating m^6^A writers and erasers in NP cells treated with IL‐1β, as analyzed by RNA‐seq. (B) Protein levels of ALKBH5, FTO, METTL3, METTL14, and WTAP in NP cells after IL‐1β treatment over time, one‐way ANOVA with Tukey‐Kramer test. (C) Typical IF images of WTAP in NP cells with or without IL‐1β treatment, two‐tailed unpaired *t*‐test. (Scale bar = 20 µm) (D) Relative m^6^A level of ACSL4 by MeRIP‐qPCR in IL‐1β‐treated NP cells with either NC or WTAP knockdown, two‐way ANOVA with Tukey‐Kramer test. (E) Relative level of ACSL4 mRNA by qRT‐PCR in shNC or shWTAP NP cells following IL‐1β stimulation, two‐way ANOVA with Tukey‐Kramer test. (F) Western blotting of WTAP and ACSL4 in IL‐1β‐challenged NP cells with WTAP knockdown or not, two‐way ANOVA with Tukey‐Kramer test. (G) Western blotting of mature‐CTSB, mature‐CTSD, LC3‐I, LC3‐II, p62, and BNIP3 in IL‐1β‐challenged NP cells with WTAP silencing or not, two‐way ANOVA with Tukey‐Kramer test. Values are shown as mean ± SD. ^*^ vs shNC, ^**^ *p* < 0.01, ^***^ *p* < 0.001; # vs shNC+IL‐1β, #p < 0.05, ##p < 0.01, ###p < 0.001. (H) Typical confocal microscopy images of NP cells transfected with mCherry‐LGALS3 plasmids, then infected with GFP‐labeled NC or WTAP knockdown lentivirus, and finally stimulated with IL‐1β for 72 h, two‐tailed unpaired *t*‐test. (Scale bar = 5 µm) (I) Super‐resolution microscopy images of Mitotracker staining (100 nM, 30 min) in IL‐1β‐treated NP cells with WTAP knockdown or not, two‐tailed unpaired *t*‐test. (Scale bar = 10 µm) (J) Typical confocal microscopy images of NP cells infected with GFP‐labeled lentivirus for NC or WTAP knockdown. Cells were co‐transfected with mCherry‐LGALS3 and either vector control or Flag‐ACSL4 overexpression plasmids, prior to IL‐1β stimulation for 72 h, two‐way ANOVA with Tukey‐Kramer test. (Scale bar = 10 µm) Values are expressed as mean ± SD. ^*^ vs shNC+IL‐1β, ^***^ *p* < 0.001; # vs shWTAP+Vector+IL‐1β, ###p < 0.001. (K) Western blotting of mature‐CTSB, mature‐CTSD, LC3‐I, LC3‐II, p62, BNIP3, and WTAP in NP cells treated as described in (J).

### 2.5 IGF2BP2 Enhances ACSL4 mRNA Stability Through m^6^A Modification

A prior study showed that “reader” proteins such as YTHDF2/3 and IGF2BP1/2/3 influence the stability of m^6^A‐modified RNA. We conducted an RNA pull‐down assay followed by mass spectrometry to pinpoint the reader that recognizes WTAP. The results indicated a strong interaction between m^6^A‐modified ACSL4 mRNA and IGF2BP2, which was further confirmed by western blot (Figure 5A–C). Knockdown of IGF2BP2 in NPCs using three specific lentiviral shRNAs suppressed IL‐1β‐induced ACSL4 expression (Figure 5D). We further performed an actinomycin D chase assay to evaluate ACSL4 mRNA stability in IL‐1β‐treated NPCs with or without IGF2BP2 knockdown. The findings revealed a significant reduction in the half‐life of ACSL4 mRNA in the shIGF2BP2 group relative to the shNC group, suggesting that IGF2BP2 depletion adversely affects ACSL4 mRNA stability (Figure 5E). To dissect the regulatory role of m^6^A modification in ACSL4 expression, we further analyzed the MeRIP‐Seq data and identified an m^6^A peak in the exon region of ACSL4, ranging from sites 109 644,109–109,643,984 (Figure 5F). We subsequently mutated two potential m^6^A sites (1947 and 1985) in the coding region to guanosine (G) and observed that IGF2BP2 silencing reduced wild‐type ACSL4 expression but did not affect the mutant ACSL4 (Figure 5G,H). Moreover, IGF2BP2 knockdown partially attenuated IL‐1β‐induced LMP and rescued the impairment of mitophagy flux (Figure 5I). Collectively, these findings suggested that IGF2BP2 mediated the enhanced stability of m^6^A‐modified ACSL4 mRNA.

**FIGURE 5:**
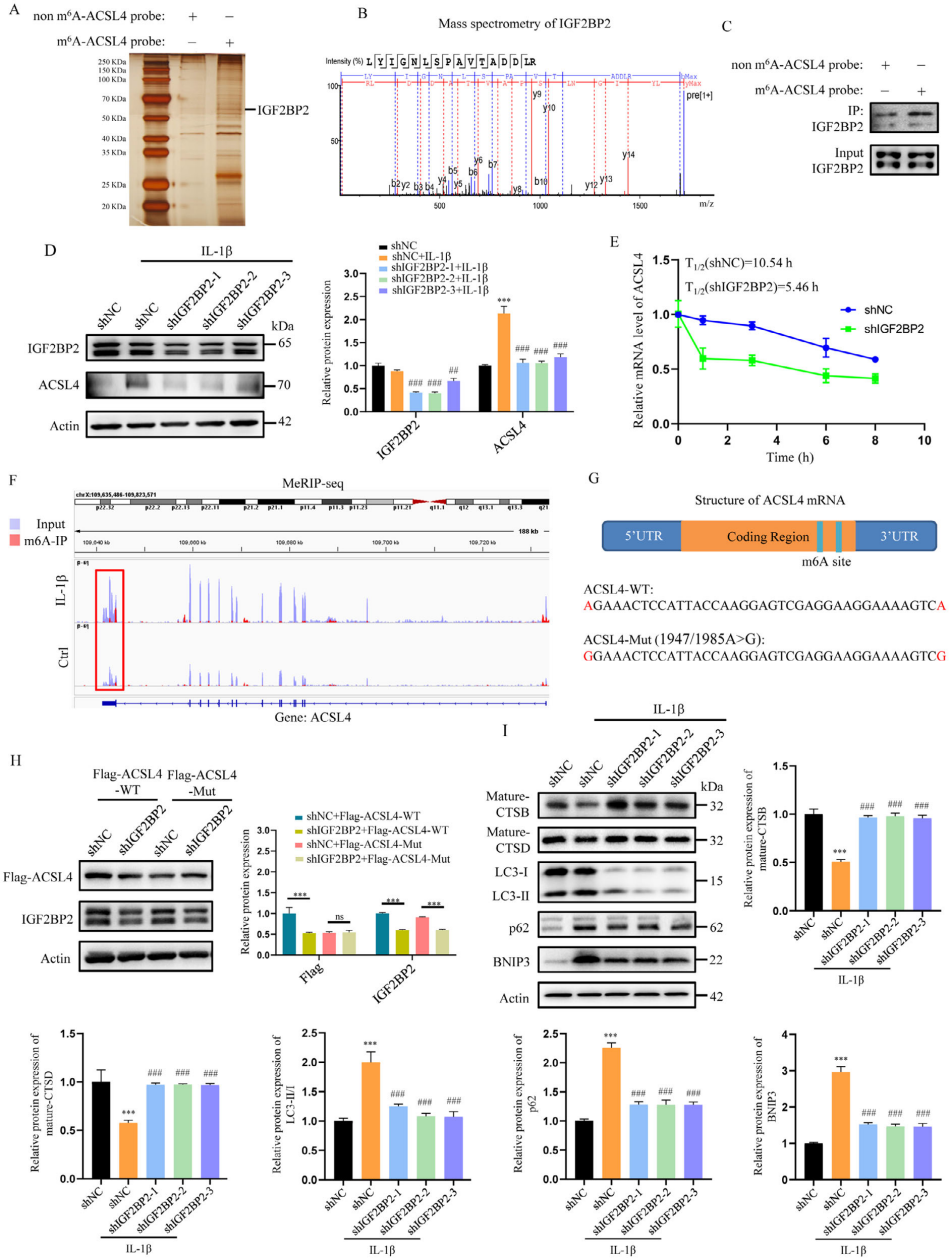
IGF2BP2 increases the stability of ACSL4 mRNA with m^6^A modification. (A) Silver staining of proteins interacting with m^6^A‐modified ACSL4 mRNA through RNA pull‐down assay. (B) Mass spectrometry analysis of IGF2BP2 protein captured by RNA pull‐down assay. (C) Confirmation of the recognition of IGF2BP2 with m^6^A‐modified ACSL4 mRNA by RNA pull‐down assay. (D) Western blotting of IGF2BP2 and ACSL4 protein levels in IL‐1β‐treated NP cells with IGF2BP2 knockdown or not, two‐way ANOVA with Tukey‐Kramer test. (E) NC and IGF2BP2 knockdown NP cells were challenged with actinomycin D (5 µg/mL), and the ACSL4 mRNA levels were evaluated by qRT‐PCR at the indicated time. The half‐life of ACSL4 mRNA was calculated by non‐linear regression fitting, with values of 10.97 h (95% CI: 8.58–13.36 h) for shNC and 6.14 h (95% CI: 1.81–10.48 h) for shIGF2BP2. (F) Integrative genomics viewer (IGV) tracks showed the m^6^A peak distribution on ACSL4 gene from MeRIP‐seq data in control and IL‐1β‐treated NPCs. (G) Construction of wild‐type (WT) and mutant (Mut, 1947/1985A>G) Flag‐ACSL4 plasmids. (H) Western blotting analysis of Flag‐ACSL4 (WT and Mut) and IGF2BP2 protein levels in NP cells with or without IGF2BP2 knockdown, two‐way ANOVA with Tukey‐Kramer test. (I) Western blotting of mature‐CTSB, mature‐CTSD, LC3‐I, LC3‐II, p62, and BNIP3 in IL‐1β‐treated NP cells with IGF2BP2 silencing or not, two‐way ANOVA with Tukey‐Kramer test. Values are expressed as mean ± SD. ^*^ vs shNC, ^***^ *p* < 0.001; # vs shNC+IL‐1β, ##p < 0.01, ###p < 0.001.

### 2.6 WTAP and ACSL4 Mediate Lysosomal Membrane Permeabilization via Lipid Peroxidation

ACSL4 catalyzes the esterification of CoA with long‐chain polyunsaturated fatty acids (PUFAs) like arachidonic acid (AA) and adrenic acid (AdA), facilitating fatty acid peroxidation or phospholipid biosynthesis. Lipid bilayer peroxidation leads to the folding of hydrophobic tails toward the aqueous phase, resulting in loss of membrane stability and increased membrane permeability. To determine whether ACSL4 localizes in proximity to lysosomes, we performed immunofluorescence co‐staining of ACSL4 with the lysosomal marker LAMP1 in NP cells. Under normal conditions, partial but not predominant co‐localization of ACSL4 with LAMP1 was observed. IL‐1β treatment increased the protein level of ACSL4 and markedly enhanced its co‐localization with LAMP1, suggesting that a subset of ACSL4 associates with the lysosomal periphery or lysosome‐associated membranes (Figure S5). Lipid peroxidation levels in NP cells were assessed using the peroxidation‐sensitive fluorescent dye BODIPY 581/591 C11. Following IL‐1β treatment, BODIPY was readily oxidized, while silencing of WTAP or ACSL4 significantly restored its non‐oxidized state (Figure 6A,B). Given that 4‐Hydroxy‐2‐nonenal (4‐HNE) is a recognized by‐product of lipid peroxidation, we investigated its accumulation in NPCs lysosomes. IL‐1β treatment increased lysosomal 4‐HNE levels, whereas suppression of WTAP or ACSL4 expression evidently ameliorated this effect (Figure 6C,D). Additionally, we measured malondialdehyde (MDA), a natural product of lipid peroxidation widely used as an oxidative stress marker. Deficiency of WTAP or ACSL4 strongly suppressed MDA formation in IL‐1β‐stimulated NPCs (Figure 6E). Furthermore, lipidomic analysis of IL‐1β‐treated NPCs with or without ACSL4 knockdown revealed that AA‐containing phospholipids, preferred substrates for oxidation, were significantly reduced upon ACSL4 silencing. Specifically, levels of AA‐containing phosphatidylethanolamine (PE) (C17:0/C20:4) and phosphatidylcholine (PC) (P‐C18:4/C20:4) were notably decreased (Figure 6F,G). Overall, our data indicated that WTAP and ACSL4 deficiency led to a significant and selective reduction in AA‐containing PE and PC species, which serve as key substrates promoting LMP.

**FIGURE 6:**
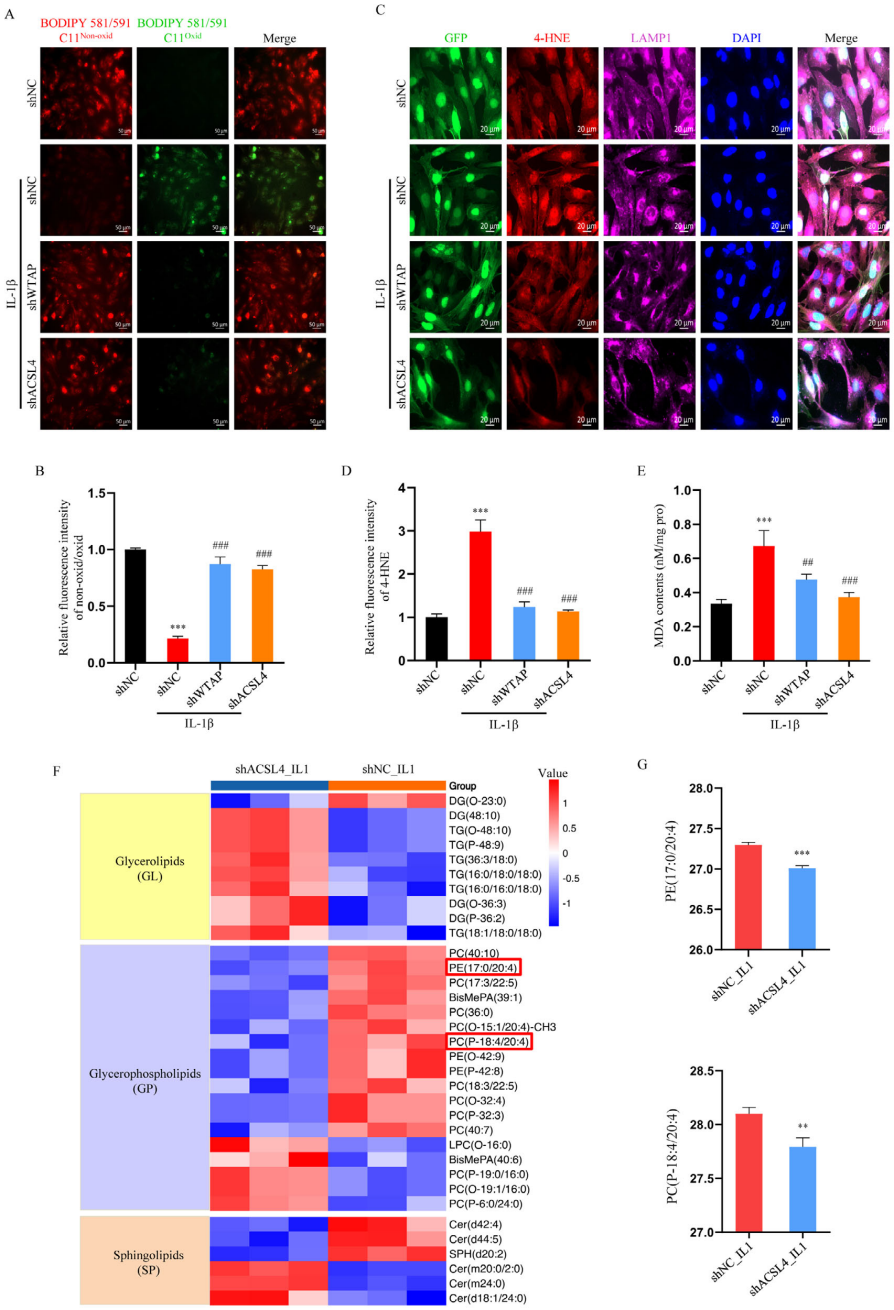
WTAP and ACSL4 mediate lysosomal membrane permeabilization via lipid peroxidation. (A, B) Representative fluorescent images of BODIPY 581/591 C11 staining in shNC, shWTAP, or shACSL4 NP cells after IL‐1β stimulation. (Red signifies non‐oxidized, and green signifies oxidized; two‐way ANOVA with Tukey‐Kramer test. Scale bar = 50 µm) (C, D) Typical images of IF staining for 4‐HNE and LAMP1 in shNC, shWTAP, or shACSL4 NP cells after IL‐1β stimulation, two‐way ANOVA with Tukey‐Kramer test. (Scale bar = 20 µm) (E) Intracellular MDA levels in NP cells with NC, WTAP or ACSL4 silencing under IL‐1β treatment, two‐way ANOVA with Tukey‐Kramer test. (F) Heat map depicting the differentially produced lipids in IL‐1β‐treated NP cells with or without silencing ACSL4. (G) The relative levels of PE(17:0/20:4) and PC(P‐18:4/20:4) in IL‐1β‐treated NP cells with or without ACSL4 knockdown, two‐tailed unpaired *t*‐test. Values are expressed as mean ± SD. ^*^ vs shNC, ^***^ *p* < 0.001; # vs shNC+IL‐1β, ##p < 0.01, ###p < 0.001.

### 2.7 Inhibition of the WTAP/ACSL4 Axis Attenuates IVDD by Restoring Lysosomal Function and Mitophagy Flux

To investigate the role of the WTAP/ACSL4 axis in IVDD, we established a rat IVDD model and administered adeno‐associated virus 9 (AAV9) with shWTAP into NP tissues via intradiscal injection (Figure 7A). The decreased expression of WTAP and ACSL4 confirmed the knockdown efficiency of AAV9 infection (Figure 7B and Figure S6A, B). Magnetic resonance imaging (MRI) analysis revealed a pronounced reduction in intervertebral disc height and signal intensity following IVDD surgery, which was significantly alleviated in the AAV9‐shWTAP group (Figure 7C and Table S2). Histological evaluation was conducted using Hematoxylin‐Eosin (H&E), Safranin O and Fast Green (SO&FG), and Alcian Blue (AB) staining methods. H&E staining demonstrated that the NP structure remained intact with tightly arranged chondrocytes in control rats, while the IVDD group exhibited severe NP disruption and chondrocyte loss (Figure 7D and Table S3). These degenerative changes were markedly attenuated upon WTAP knockdown. The protective effect was further corroborated by SO&FG and AB staining (Figure 7D). Immunohistochemical analysis of collagen II and matrix metalloproteinase 13 (MMP13) revealed that WTAP silencing enhanced extracellular matrix (ECM) synthesis in the NP and mitigated ECM degradation in IVDD rats (Figure 7E and Figure S6C,D). Staining for cathepsins (CTSB and CTSD), LC3, p62, and BNIP3 indicated that WTAP deficiency partially restored lysosomal function and mitophagy flux in NP tissues (Figure 7F–I and Figure S6E–I). Moreover, 4‐HNE staining demonstrated elevated lipid peroxidation in IVDD models, which was reduced following WTAP knockdown (Figure 7J and Figure S6J). These results demonstrated that inhibition of the WTAP/ACSL4 axis could delay IVDD progression by restoring lysosomal function and mitophagy flux.

**FIGURE 7:**
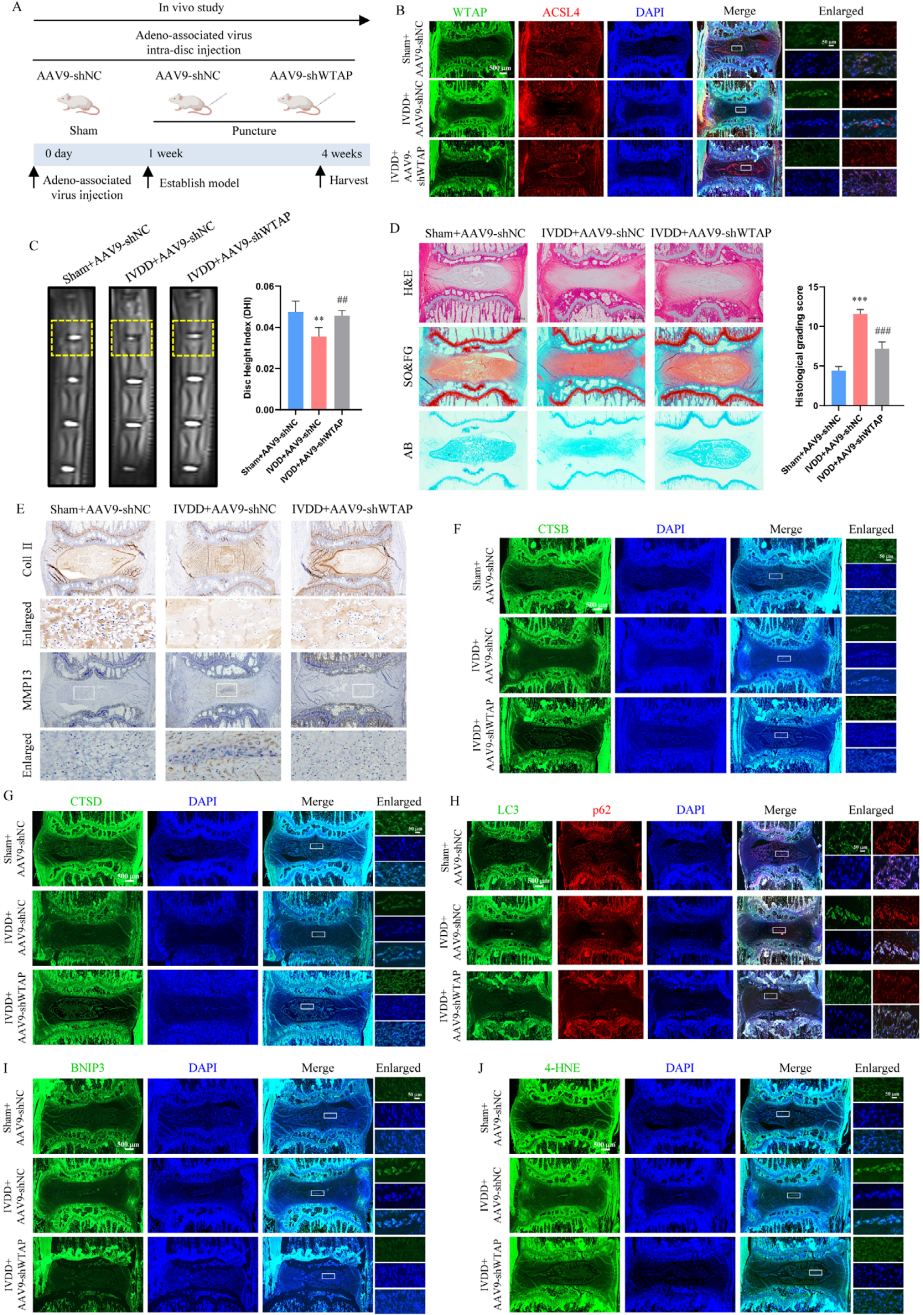
Inhibition of the WTAP/ACSL4 axis attenuates IVDD by restoring lysosomal function and mitophagy flux. (A) Schematic depicting the establishment of the IVDD model and the experimental protocol designed to assess the in vivo effects of WTAP knockdown. (B) Representative IF images of WTAP and ACSL4 in rat tail IVD tissues in the sham+AAV9‐shNC, IVDD+AAV9‐shNC, and IVDD+AAV9‐shWTAP groups. (Scale bar = 500 µm, Enlarged scale bar = 50 µm) (C) MRI images and Disc Height Index (DHI) of rat caudal intervertebral discs across groups, two‐way ANOVA with Tukey‐Kramer test. (D) Histological grading scores of rat tail IVD tissues by H&E, SO&FG and AB staining, two‐way ANOVA with Tukey‐Kramer test. (Scale bar = 500 µm) (E) IHC analysis of collagen II and MMP13 protein levels in rat tail IVD tissues. (Scale bar = 500 µm, Enlarged scale bar = 50 µm) (F, G) Typical IF images of CTSB and CTSD in rat tail IVD tissues from each group. (Scale bar = 500 µm, Enlarged scale bar = 50 µm) (H) Representative IF images showing LC3 co‐localized with p62 in rat tail IVD tissues. (Scale bar = 500 µm, Enlarged scale bar = 50 µm) (I, J) Typical IF images of BNIP3 and 4‐HNE in rat tail IVD tissues. (Scale bar = 500 µm, Enlarged scale bar = 50 µm) Values are presented as mean ± SD. ^*^ vs sham+AAV9‐shNC, ^*^ *p* < 0.05, ^**^ *p* < 0.01 and ^***^ *p* < 0.001; # vs IVDD+AAV9‐shNC, ##p < 0.01, ###p < 0.001.

## 3 Discussion

IVDD is a multifactorial disorder characterized by NP cell dysfunction, ECM degradation, and inflammatory microenvironment dysregulation. Accumulating evidence highlights the critical role of impaired autophagy in IVDD progression; however, the upstream regulatory mechanisms remain incompletely delineated. This study conclusively demonstrates that LMP is crucial in IVDD pathogenesis and that its targeted inhibition effectively mitigates IVDD in vivo. Furthermore, we identify the methyltransferase WTAP as a key upstream regulator, significantly upregulated in IL‐1β‐stimulated NP cells and degenerated IVD tissues, where it acts as a critical inducer of LMP. Mechanistically, WTAP promotes m^6^A modification and stabilization of ACSL4 mRNA in an IGF2BP2‐dependent manner. Elevated ACSL4 promotes lipid peroxidation, a key driver of lysosomal membrane destabilization that culminates in LMP. Consistently, disrupting either WTAP or ACSL4 rescues IL‐1β‐induced LMP, restores mitophagic flux, and alleviates IVDD progression, highlighting the therapeutic potential of targeting this axis (Figure 8).

**FIGURE 8:**
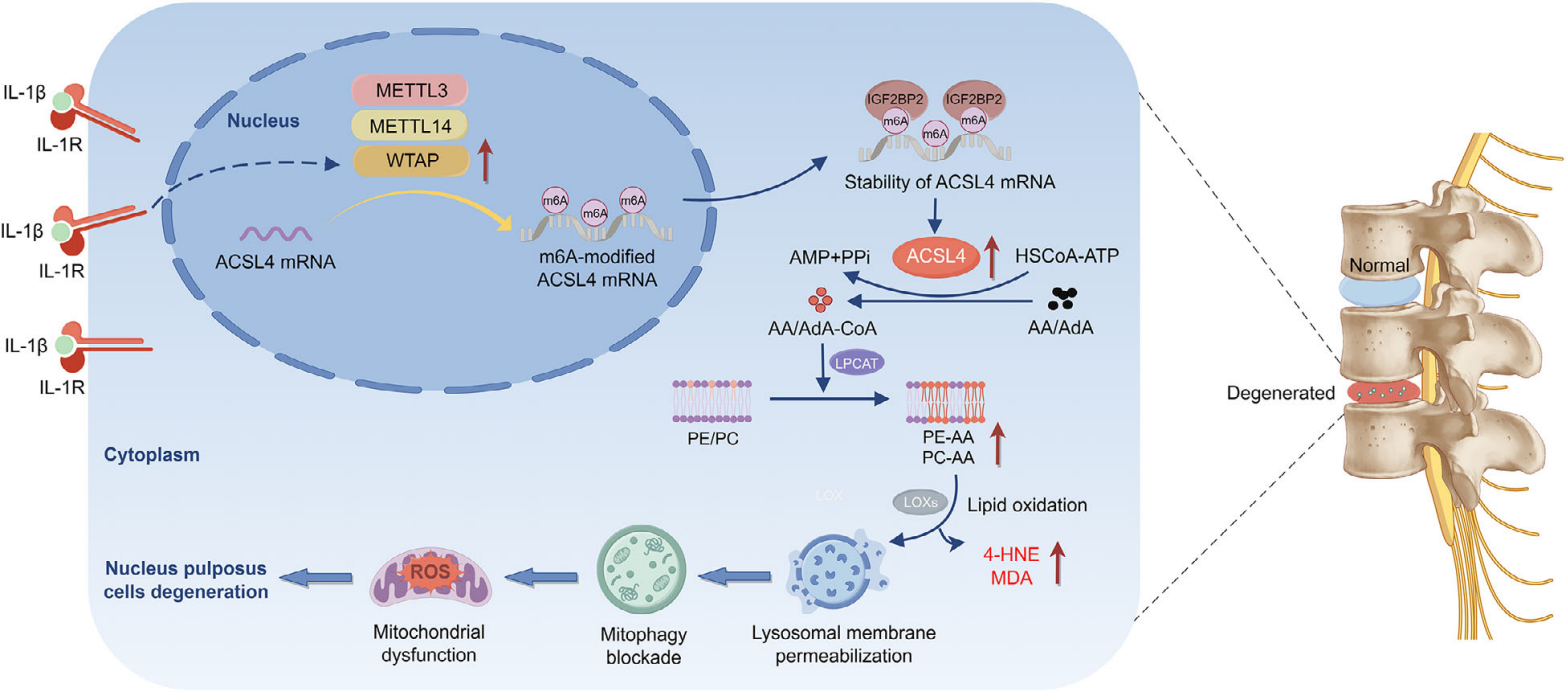
Diagram illustrating the role of the WTAP‐ACSL4 axis in NPC lysosomal membrane permeabilization and IVDD progression. IL‐1β upregulates the protein level of WTAP, which promotes the m^6^A modification of ACSL4 mRNA in an IGF2BP2‐dependent manner, thus enhancing its stability. The increased ACSL4 drives lipid peroxidation, leading to lysosomal membrane permeabilization (LMP) and impaired mitophagy, which collectively accelerate intervertebral disc degeneration (IVDD). Notably, therapeutic targeting of either WTAP or ACSL4 effectively rescues LMP and mitigates IVDD progression, highlighting a promising intervention strategy.

Lysosomes serve as the final executors of the autophagic degradation process, and dysfunction or deficiency of lysosomes has been implicated in various tissue degeneration and autophagy‐related diseases [24, 25, 26]. Our previous research showed that oxidative stress‐triggered LMP contributes to IVDD pathology by compromising lysosomal autophagic function, thereby exacerbating NP cell senescence [13]. In addition, overloading has also been shown to trigger LMP, leading to impaired autophagic flux and NP cell death [14]. This study's integrated single‐cell transcriptomic and proteomic analyses of human NP tissues identified altered lysosomal pathway enrichment and reduced mature cathepsins (CTSB/CTSD) levels as hallmarks of severe degeneration. The decreased colocalization of CTSB/CTSD with LAMP1, coupled with direct observation of lysosomal membrane rupture via TEM, confirmed LMP occurrence in IVDD. Mitophagy, a conserved process to degrade defective mitochondria, constitutes a prerequisite for maintaining NP cell homeostasis [27, 28]. We observed an accumulation of mitophagy‐related proteins (elevated LC3‐II/I ratio, p62, PINK1, and BNIP3) in severely degenerated human NP tissues. Notably, LMP induction by LLOME recapitulated this mitophagy flux impairment (elevated LC3‐II/I ratio, p62, and BNIP3), establishing LMP as a direct driver of impaired mitophagy in NP cells. To further establish the causal relationship between IL‐1β‐induced LMP and mitophagic dysfunction under pathological conditions, we employed a rescue strategy by overexpressing TFEB, a master regulator of lysosomal biogenesis [29]. Our data demonstrated that Flag‐TFEB overexpression significantly reduced IL‐1β‐induced LGALS3 puncta formation and restored autophagic flux, as evidenced by decreased LC3‐positive yellow puncta (mCherry^+^/GFP^+^) and reduced accumulation of LC3‐II/I, p62, and BNIP3 (Figure S7). These results provide direct causal evidence that IL‐1β‐induced LMP is a primary upstream event driving mitophagic impairment in NP cells.

Single‐cell transcriptomics revealed the infiltration of M1 and M2 macrophages into NP tissue during IVDD [30, 31, 32]. NP cells are subjected to a persistent inflammatory environment enriched in cytokines, including IL‐1β, IL‐6, and TNF‐α. We found sustained IL‐1β stimulation dysregulated ECM homeostasis, decreasing collagen II and increasing MMP13 expression in NP cells. Activation of NLRP3 inflammasome protein complexes contributes to the maturation and release of IL‐1β. Lysosomal membrane rupture leads to the leakage of internal cathepsins (such as cathepsin B) into the cytoplasm, activating the NLRP3 inflammasome and facilitating IL‐1β maturation [33, 34]. However, the reciprocal impact of chronic IL‐1β exposure on lysosomal integrity in NP cells remains elusive. Here, we identify IL‐1β as a potent inducer of LMP in NP cells. IL‐1β treatment led to lysosomal membrane rupture, evidenced by LGALS3 puncta formation and TEM observation, impaired cathepsin maturation, and subsequent defective mitophagic flux. This mitophagy impairment resulted in the accumulation of defective mitochondria, demonstrated by mitochondrial depolarization and elevated ROS in NP cells. These findings delineate a clear pathogenic pathway from inflammatory stimulation to LMP and subsequent mitochondrial dysfunction in IVDD.

N6‐methyladenosine (m^6^A) modification is a critical epigenetic regulator of gene expression in numerous diseases, such as IVDD [35, 36, 37, 38]. Our MeRIP‐Seq analysis identified ACSL4 as a key m^6^A‐modified target in IL‐1β‐stimulated NP cells. Functional validation confirmed that ACSL4 upregulation mediates both LMP and mitophagy impairment. ACSL4, an acyl‐CoA synthetase that preferentially activates PUFAs, is known to promote ferroptosis via lipid peroxidation [39, 40], but its role in lysosomal function remains unreported. Our lipidomic and biochemical analyses indicated that ACSL4 knockdown reduces lipid peroxidation markers (4‐HNE, MDA) and AA‐containing phospholipids (PE C17:0/C20:4, PC P‐C18:4/C20:4), which are critical substrates for membrane lipid peroxidation. Lysosomes are enriched in cholesterol and sphingolipids (such as sphingomyelin), which reduce the fluidity of the membrane, protect against internal hydrolytic enzymes, and limit the passive leakage of protons [41]. In contrast, PUFAs such as AdA and AA introduce kinks into phospholipid tails of lysosomal membrane, increasing fluidity and permeability. Their incorporation, especially when peroxidized, renders lysosomal membranes more susceptible to hydrolysis and rupture [42]. Therefore, IL‐1β enhances the susceptibility of NP cells to LMP via ACSL4‐mediated lipid peroxidation, and ACSL4 inhibition represents a promising strategy to counteract IVDD under inflammatory conditions.

Furthermore, we identified WTAP as the methyltransferase responsible for ACSL4 m^6^A modification. While METTL3 serves as the core catalytic subunit, WTAP functions as a regulatory subunit that recruits the m^6^A methyltransferase complex to specific mRNAs. Specifically, WTAP is essential for METTL3 and METTL14 localization and accumulation in nuclear speckles [43]. WTAP is known to influence cell proliferation and ECM synthesis in NP cells [23], yet its involvement in lysosomal‐mitochondrial crosstalk remains unexplored. Our data demonstrated that WTAP silencing rescues IL‐1β‐induced LMP and mitophagy flux, and attenuates IVDD progression in vivo. Knockdown of WTAP reduces the upregulation of lipid peroxidation markers 4‐HNE and MDA induced by IL‐1β. This is consistent with recent findings that WTAP promotes lipid metabolism dysregulation in non‐alcoholic steatohepatitis [44], suggesting a conserved role of WTAP in regulating ACSL4‐mediated lipid peroxidation across diseases. Additionally, we identified IGF2BP2 as the m^6^A reader that stabilizes ACSL4 mRNA, as IGF2BP2 knockdown shortened ACSL4 mRNA half‐life and abrogated its upregulation. This extends the known function of IGF2BP2 in promoting mRNA stability to the regulation of lysosomal and mitochondrial homeostasis in IVDD. Collectively, our study identifies the WTAP/m^6^A/IGF2BP2/ACSL4/lipid peroxidation axis as a new regulatory pathway driving LMP and mitophagy flux impairment in IVDD.

To confirm the specificity of the WTAP/ACSL4 axis in this process, we performed epistasis rescue experiments. Knockdown of WTAP significantly alleviated IL‐1β‐induced LMP and mitophagic flux impairment, as reflected by reduced LGALS3 puncta, restored mature cathepsin B/D levels, and decreased LC3‐II/I and p62 accumulation. Strikingly, forced overexpression of ACSL4 effectively reversed these protective effects, re‐inducing LGALS3 puncta formation, reducing mature cathepsin levels, and re‐accumulating mitophagy markers (Figure 4J,K and Figure S4). These experiments demonstrate that ACSL4 is a key downstream effector of WTAP in controlling LMP and mitophagic dysfunction. Additionally, the in vivo validation using AAV9‐mediated WTAP silencing provides strong preclinical evidence for targeting the WTAP/ACSL4 axis in IVDD. WTAP knockdown restored lysosomal function (elevated mature CTSB/CTSD), mitophagic flux (reduced LC3‐II/I and p62), and ECM synthesis (increased collagen II, decreased MMP13), leading to improved disc height and structure. Notably, lipid peroxidation inhibitors (e.g., ferrostatin‐1) have been shown to alleviate IVDD [45]. Combining m^6^A‐targeted therapies (e.g., WTAP inhibitors) with lipid peroxidation modulators may provide a synergistic approach for IVDD treatment.

This study still has several limitations. Although we conducted lipidomic analysis on human primary NP cells treated with IL‐1β, metabolomic profiling of human IVD tissues across various Pfirrmann grades is necessary to directly link metabolic changes with degeneration. Culturing NP cells in 10% FBS can modify their phenotype relative to the native state in IVD tissue. Future research will utilize low‐serum or serum‐free media with added growth factors to more accurately replicate the physiological conditions of IVD tissue. Third, for the mRNA stability assay, we only collected samples at time points up to 8 h after actinomycin D treatment. Although the exponential decay model fitted the observed data well and enabled reliable half‐life estimation via non‐linear regression, detection at longer time points would further improve the accuracy and robustness of half‐life calculation. Future work will include extended time points to further validate the degradation kinetics of ACSL4 mRNA. Fourth, the molecular mechanism underlying IL‐1β‐induced WTAP upregulation remains unclear and warrants further investigation. Although our in vitro rescue experiments demonstrate that WTAP regulates LMP and mitophagy through ACSL4, this study lacks in vivo genetic rescue data to confirm that WTAP knockdown ameliorates disc degeneration specifically via ACSL4. Future studies involving co‐delivery of AAV‐shWTAP and AAV‐ACSL4 in animal models are warranted to further validate the translational potential of targeting this axis.

In conclusion, our study reveals a novel pathogenic pathway in IVDD, where the inflammatory environment enhances WTAP‐mediated m^6^A modification of ACSL4 mRNA, stabilized by IGF2BP2. Elevated ACSL4 drives lipid peroxidation, leading to LMP. The resultant lysosomal dysfunction impairs mitophagic flux, causing the accumulation of damaged mitochondria and fostering cellular degeneration. This WTAP/ACSL4/LMP/mitophagy axis represents a promising therapeutic target for intervening in the progression of IVDD.

## 4 Materials and Methods

### 4.1 Ethics Approval and Consent to Participate

From patients with Pfirrmann grade II (mild) or grade IV (severe) disc degeneration, nucleus pulposus samples were obtained during percutaneous endoscopic lumbar discectomy (PELD) procedures. Inclusion criteria were: age 18–70 years; persistent low back and/or radicular pain for at least 3 months unresponsive to conservative management; and surgical indication consistent with the degenerated level. Exclusion criteria were: previous surgery at the same level; spinal infection, tumor, fracture, or inflammatory arthritis; severe osteoporosis; autoimmune disease; active systemic infection; or pregnancy. Table S4 provides comprehensive sample information. Primary nucleus pulposus cells were isolated and cultured according to the previously described protocols [46]. All experimental procedures, including the rat studies, were ethically approved by the Ethics Committee of the Affiliated Hospital of Jining Medical University (Approval nos. 2021C159 and 2021C119).

### 4.2 Quality Control and Processing of Single‐Cell RNA Sequencing Data

Single‐cell RNA‐seq analysis was performed based on a gene expression matrix from our prior dataset (GSE251686) and an independent dataset (GSE153066, 8 control and 8 IVDD patients) of human NP tissues. Raw data processing, including barcode demultiplexing, read alignment, and UMI quantification, was conducted using the CellID pipeline on the Celelens cloud platform. A filtering procedure was carried out to remove low‐quality cells, based on three criteria: a minimum of 200 expressed genes, a maximum mitochondrial percentage of 20%, and a per‐sample upper cutoff (top 2%) for gene or UMI counts. The DoubletFinder algorithm was utilized to detect and remove potential cell doublets. The matrix was subjected to normalization and log transformation, after which feature selection was performed to identify highly variable genes for subsequent dimension reduction. Principal component analysis (PCA) was implemented, followed by Harmony‐based batch effect correction to remove technical variation among samples. Cell clustering was conducted based on batch‐corrected embeddings, and cell types were identified by the expression signatures of classic marker genes.

### 4.3 Cell Transfection

Lentiviruses carrying shRNA targeting WTAP, ACSL4, and IGF2BP2 (constructed with the GV493 vector) were purchased from Genechem Inc. Table S5 shows the shRNA sequences. NP cells at 30% confluence were infected with virus at an MOI of 100 using HiTransG P reagent. Wild‐type and mutant (1947/1985A>G) human ACSL4 plasmids were constructed by Genechem Inc. using the GV657 vector, which were transfected into NP cells via Lipofectamine 3000.

### 4.4 Transmission Electron Microscopy

After three washes with pre‐warmed phosphate‐buffered saline (PBS), NP cells were initially fixed with a mixture of 4% paraformaldehyde and 2.5% glutaraldehyde in PBS for 2 min. The fixed cells were gently scraped, pelleted by centrifugation, and washed thrice with PBS. Post‐fixation was conducted in 1% osmium tetroxide for 1 h, and the samples were dehydrated via a stepped ethanol gradient (50%‐100%) and absolute acetone. Infiltration was carried out with an acetone‐epoxy resin mixture (1:3) for 3 h, after which the specimens were embedded in resin and polymerized at 60°C overnight. Sections (70–90 nm) were loaded onto copper grids and post‐stained with uranyl acetate and lead citrate for improved contrast. Lysosomal ultrastructure was visualized under a Talos L120C transmission electron microscope (Thermo Fisher Scientific).

### 4.5 Methylated RNA Immunoprecipitation Sequencing (MeRIP‐seq)

RNA was purified from three independent batches of NP cells with TRIzol. Following confirmation of RNA integrity (RIN > 7.0), mRNA was captured with the Dynabeads mRNA DIRECT Purification Kit and subsequently fragmented with the Magnesium RNA Fragmentation Module. Fragmented RNA underwent m^6^A immunoprecipitation by incubating with an anti‐m^6^A antibody in IP buffer at 4 °C for 2 h. This was followed by capture using protein A/G magnetic beads and subsequent washing with low‐ and high‐salt buffers. The RNA obtained from immunoprecipitation was eluted and prepared for library construction via the NEBNext Ultra II RNA Library Prep Kit. Libraries underwent quality assessment using a Qsep100 analyzer and were subjected to 2×150 bp paired‐end sequencing on an Illumina NovaSeq 6000 platform. Reads were processed with Bowtie2 for alignment to the GRCh38/hg38 genome, and uniquely mapped reads were selected with SAMtools for data analysis.

### 4.6 MeRIP‐qPCR

The EpiQuik CUT&RUN m^6^A RNA Enrichment Kit was used for MeRIP. In brief, NP cells were disrupted with the provided lysis buffer. The anti‐m^6^A antibody was immobilized on magnetic beads and incubated with cell lysate under rotation for 2 h. Following incubation, the supernatant was discarded, and the RNA complexes bound to the beads were digested using proteinase K buffer. RNA was subsequently extracted using a phenol‐chloroform‐isoamyl alcohol mixture. qRT‐PCR assays were conducted for m^6^A‐modified RNA enrichment analysis, with results normalized to the input. Table S6 lists the primer sequences.

### 4.7 mRNA Stability Assays

After 72 h of lentiviral infection (NC or IGF2BP2 knockdown), NP cells were challenged with 5 µg/mL actinomycin D to suppress transcription. Total RNA was extracted at intervals of 0, 1, 3, 6, and 8 h, and ACSL4 expression was quantified via qRT‐PCR. After normalizing to the initial (0 h) level, the decay data were plotted and fitted to a one‐phase exponential decay model with GraphPad Prism 11's non‐linear regression. The decay constant (*k*) was estimated iteratively, and the half‐life was calculated as t_1/2_ = ln2/*k*. The 95% confidence intervals (CI) for half‐life were generated using the asymptotic likelihood method.

### 4.8 RNA Pull‐Down Assay

m^6^A‐modified ACSL4 RNA probes labeled with biotin, together with their non‐m^6^A controls, were prepared by Sangon Biotech (Shanghai, China). Table S7 shows the corresponding RNA oligonucleotide sequences. RNA pull‐down assays utilized the Pierce Magnetic RNA‐Protein Pull‐Down Kit. Streptavidin‐conjugated magnetic beads were used to immobilize the biotin‐labeled RNA probes. NP cell lysates (protein concentration ≥ 2 mg/mL) were prepared and incubated with RNA‐bound beads at 4°C for 1 h under gentle rotation. After incubation, the beads underwent washing, followed by elution of the RNA‐protein complexes. The eluted proteins were then analyzed by western blotting.

### 4.9 Lipidomics

NP cells, seeded at 2.5 × 10^6^ cells per 25‐cm^2^ flask, were infected with either shNC or shACSL4 lentiviruses for 24 h. 20 ng/mL IL‐1β was then added to the medium for 72‐h incubation. Post‐treatment, cells were collected and rapidly preserved at −80°C for lipidomic profiling. Lipid extraction from NP cells was performed, and lipid profiling was conducted by Oebiotech (Shanghai, China). Chromatographic separation and mass spectrometric analysis were carried out on the ACQUITY UPLC I‐Class plus system (BEH C8 column, 100×2.1 mm, 1.7 µm). Lipid classification and annotation utilized Lipid Bank (http://lipidontology.com/) and LIPID MAPS (https://www.lipidmaps.org/). Data plots were generated with the ggplot2 package (version 3.4.4). Differentially abundant lipids were those with p < 0.05 and variable importance in projection (VIP) > 1.0.

### 4.10 Establishment of Needle‐Induced Rat IVDD Model and Therapeutic Intervention

Six– to eight‐week‐old Sprague‐Dawley (SD) rats underwent AAV9 transfection experiments and were randomly separated into triplicate groups (n = 5 per group): sham+AAV9‐shNC, IVDD+AAV9‐shNC, and IVDD+AAV9‐shWTAP. Rats were anesthetized using 2.5% isoflurane inhalation and positioned prone on a 37°C heating pad. The Co6/7 disc was located through palpation and subsequently cleaned with alcohol. A micro‐injection syringe (Hamilton Bonaduz, Switzerland) was used to slowly inject 3 µL of either AAV9‐shNC or AAV9‐shWTAP (3.56×10^13^ pfu/mL) into the IVD tissue of each rat. The needle was allowed to rest for 5 s, followed by a slow extraction to minimize mechanical disturbance to the nucleus pulposus. One week after intradiscal AAV9 injection, rats underwent either sham surgery or needle puncture‐induced IVDD surgery. In the IVDD model, a 21G needle was introduced perpendicularly into the Co6/7 disc, reaching the nucleus pulposus center, rotated 180°, and held for 30 s. Post‐operatively, animals were monitored daily with free activity, standard feeding, and water access. Four weeks after surgery, the rats were sacrificed, and tail discs were collected for analysis.

### 4.11 Magnetic Resonance Imaging (MRI) Examination

At 4 weeks post‐surgery, rats in each group were anesthetized with 10% sodium pentobarbital and placed in a prone position for MRI. Disc signal intensity and structural changes were assessed using a 3.0 T MRI system (Siemens, Germany). Sagittal T2‐weighted imaging was acquired using parameters: a 1.5 mm slice thickness, repetition time (TR) of 2500 ms, and echo time (TE) of 70 ms. Quantitative analysis of Disc Height Index (DHI) was performed using ImageJ software based on the acquired MRI images. DHI was derived from the equation: DHI = [(a + b + c)/3] / [(A + B)/2], where a, b, and c represent the anterior, middle, and posterior heights of the intervertebral disc, and A and B signify the sagittal diameters of the upper and lower vertebral bodies.

### 4.12 Histological Analysis

Rats were euthanized at 4 weeks post‐surgery to collect IVD samples. After fixation in 4% paraformaldehyde for 24 h, the discs underwent decalcification with 10% EDTA over a 4‐week period. The samples were subsequently embedded in paraffin and sagittally sectioned at 5‐µm thickness, encompassing the endplate, annulus fibrosus, and nucleus pulposus regions. Disc structure was histologically evaluated via Hematoxylin‐Eosin (H&E), Alcian Blue (AB), and Safranin O and Fast Green (SO&FG) staining methods. Histological grading utilized the Masuda scoring system, assessing disc degeneration through four parameters: (I) annulus fibrosus morphology, (II) the annulus fibrosus‐nucleus pulposus border, (III) nucleus pulposus cellularity, and (IV) nucleus pulposus matrix [47]. Each parameter was scored on a 1–3 scale. The scores of the four parameters were summed to obtain a total score per disc (range 4–12), with a higher score indicating more severe degeneration.

### 4.13 Immunohistochemistry (IHC) and Immunofluorescence (IF) Staining

The rat IVD tissues were subjected to IHC staining as previously reported [13]. Primary antibodies targeting collagen II (GB115759, Servicebio) and MMP13 (GB 11247, Servicebio) were used. Following TBST washing, slides were probed using an HRP‐linked goat anti‐rabbit antibody, developed with 3,3’‐diaminobenzidine (DAB) substrate, and examined with an Axio Zoom.V16 stereomicroscope (Zeiss, Germany).

The human primary NP cells and human and rat IVD tissues underwent IF staining as previously described [48]. All slides and NP samples were exposed to primary antibodies and suitable fluorescent secondary antibodies. Imaging was performed via the Axio Zoom.V16 stereomicroscope or Zeiss LSM800 confocal laser scanning microscope. Fluorescence intensity was semiquantitatively evaluated with ImageJ software (v. 1.46r, National Institutes of Health). Antibody details are summarized in Table S8.

### 4.14 Western Blotting

The proteins of NP tissues and cells were extracted using RIPA buffer, and the concentrations were quantified via a BCA kit. Proteins were subjected to western blotting according to the previously reported method [46]. The uncropped images are available in the Supplementary original blots. Antibody details are listed in Table S8.

### 4.15 Measurement of Lipid Peroxidation

NP cells were infected with shNC, shWTAP or shACSL4 lentiviruses for 24 h, then challenged with IL‐1β (20 ng/mL) for 72 h. Afterwards, cells were incubated with 5 µM BODIPY581/591 C11 for 30 min under light‐protected conditions. Following three more PBS washes, confocal imaging (Zeiss LSM800) recorded green (oxidized, 488/510 nm) and red (non‐oxidized, 581/591 nm) signals. For each sample, five random fields were selected, and the red‐to‐green fluorescence ratio was calculated as an indicator of lipid oxidation. ImageJ software was employed for semi‐quantitative fluorescence analysis.

NP cells were plated in 6‐well plates and treated as indicated. Proteins were extracted using RIPA buffer (0.1 mL per 1 × 10^6^ cells), and the concentrations were quantified by a BCA kit. For malondialdehyde (MDA) quantification, 0.1 mL aliquots of cell supernatants were reacted with 0.2 mL of 2‐thiobarbituric acid (TBA) reagent and heated at 100°C for 15 min. The reaction mixture was equilibrated to ambient temperature. After centrifugation at 1000×g for 10 min, the supernatant was collected, and its absorbance at 532 nm was read. MDA values were calculated against a standard curve, adjusted to the protein concentration, and presented as µmol/mg protein.

### 4.16 Visualization of Mitochondrial Morphology

NP cells were placed in an 8‐well chamber slide (Cellvis, USA) and subjected to the corresponding treatments. The cells were stained with 100 nM MitoBright LT Deep Red at 37°C for 30 min in darkness. Then the cells underwent three PBS washes to eliminate residual dye. Mitochondrial imaging was performed on a Zeiss Elyra7 super‐resolution microscope (Ex 640 nm / Em 670 nm).

### 4.17 Evaluation of Mitochondrial ROS Levels and Mitochondrial Membrane Potential (MMP)

To assess mitochondrial function, MMP and mitochondrial ROS production were measured using Rhodamine 123 and MitoSox Red, respectively. Post‐stimulation, cells were incubated with 1 µM fluorescent probe at 37°C for 30 min in darkness. Following three additional washes with PBS, cells were counterstained with DAPI and then visualized on a Zeiss LSM800 confocal laser scanning microscope. Fluorescence detection was performed using Rhodamine 123 at 507/529 nm and MitoSox Red at 396/610 nm.

### 4.18 Statistical Analysis

For statistical evaluation, GraphPad Prism 11 (GraphPad Inc., La Jolla, CA, USA) was applied. Differences were tested by two‐tailed unpaired *t*‐test, one‐way ANOVA, or two‐way ANOVA with Tukey‐Kramer test as appropriate. All results correspond to mean ± SD derived from at least three independent replicates. Statistical thresholds: *p* > 0.05 = not significant (ns); *p* < 0.05 = significant (^*^ *p* < 0.05, ^**^ *p* < 0.01, ^***^ *p* < 0.001).

## Author Contributions

Conceptualization: **Shu Jia**, **Chunyang Meng**. Methodology: Shu Jia, **Xu Gao**, **Laimin Zhu**, **Haixia Song**, **Shang Chen**, **Sheng Gao**, **Lu Zhang**. Data curation: Shu Jia, Xu Gao, Laimin Zhu, **Jing Wu**, Shang Chen, Sheng Gao, Lu Zhang. Formal analysis: Xu Gao. Writing – original draft: Shu Jia. Writing – review & editing: Shu Jia, Chunyang Meng. Supervision: Shang Chen, Sheng Gao. Funding acquisition: Chunyang Meng, Shu Jia.

## Conflicts of Interest

The authors declare no conflicts of interest.

## Supporting information




**Supporting File 1**: advs77401‐sup‐0001‐SuppMat.pdf.

**Supporting File 2**: advs77401‐sup‐0001‐SuppMat.pdf.

## Data Availability

The datasets supporting the conclusions of this study are accessible from the corresponding author upon justifiable request.
