# Supplementary material for: ACSL4‐Dependent Lysosomal Lipid Peroxidation Links WTAP‐Mediated m6A Modification to Intervertebral Disc Degeneration
Source: Adv Sci (Weinh). 2026 Aug 29:e77401. Online ahead of print. doi: 10.1002/advs.77401 (PMC13525423; doi:10.1002/advs.77401)

Supplementary original blots for

ACSL4-dependent lysosomal lipid peroxidation links WTAP-mediated m6A modification to intervertebral disc degeneration

Figure. 1F

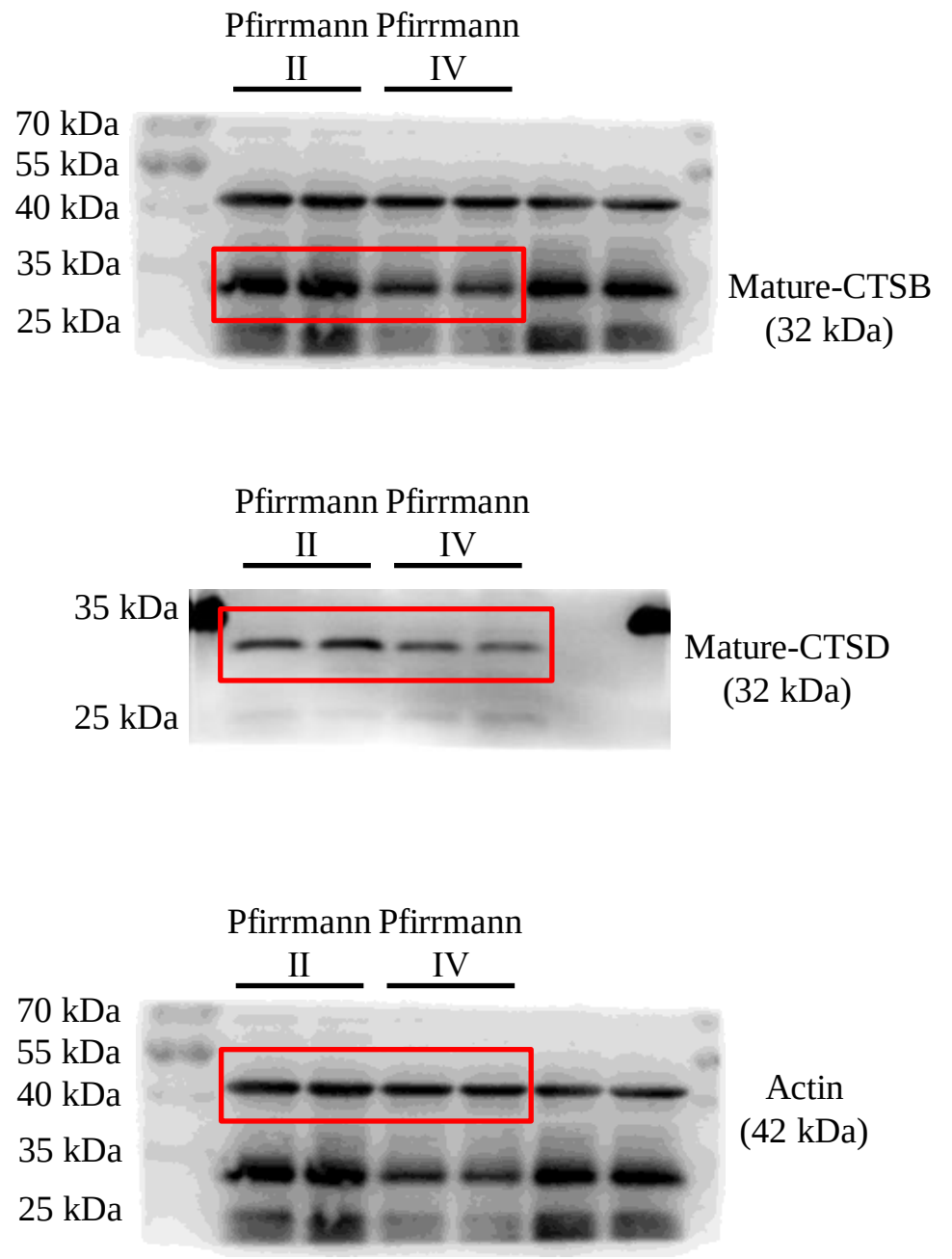

Figure. 1I

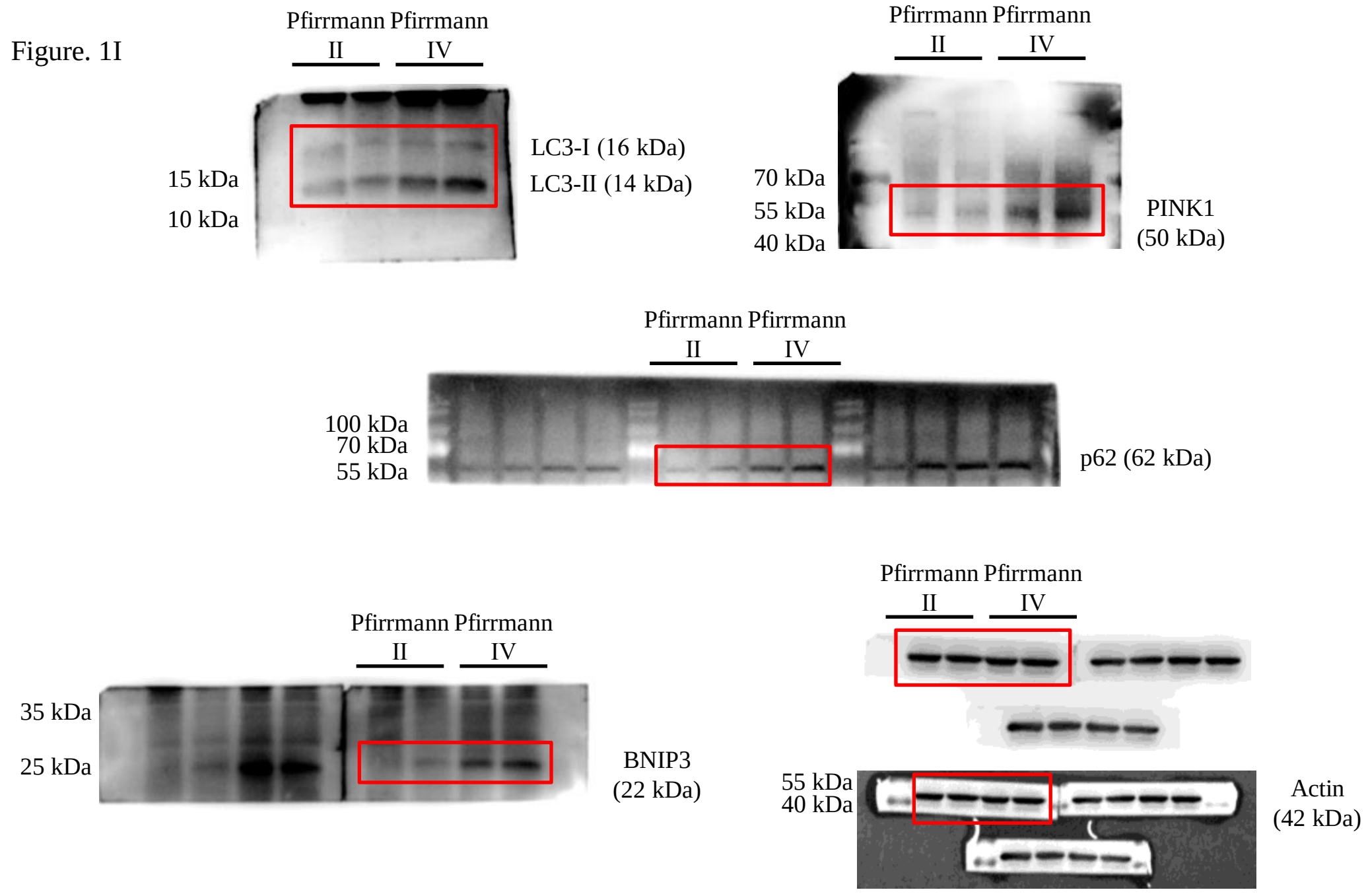

Figure. 1K

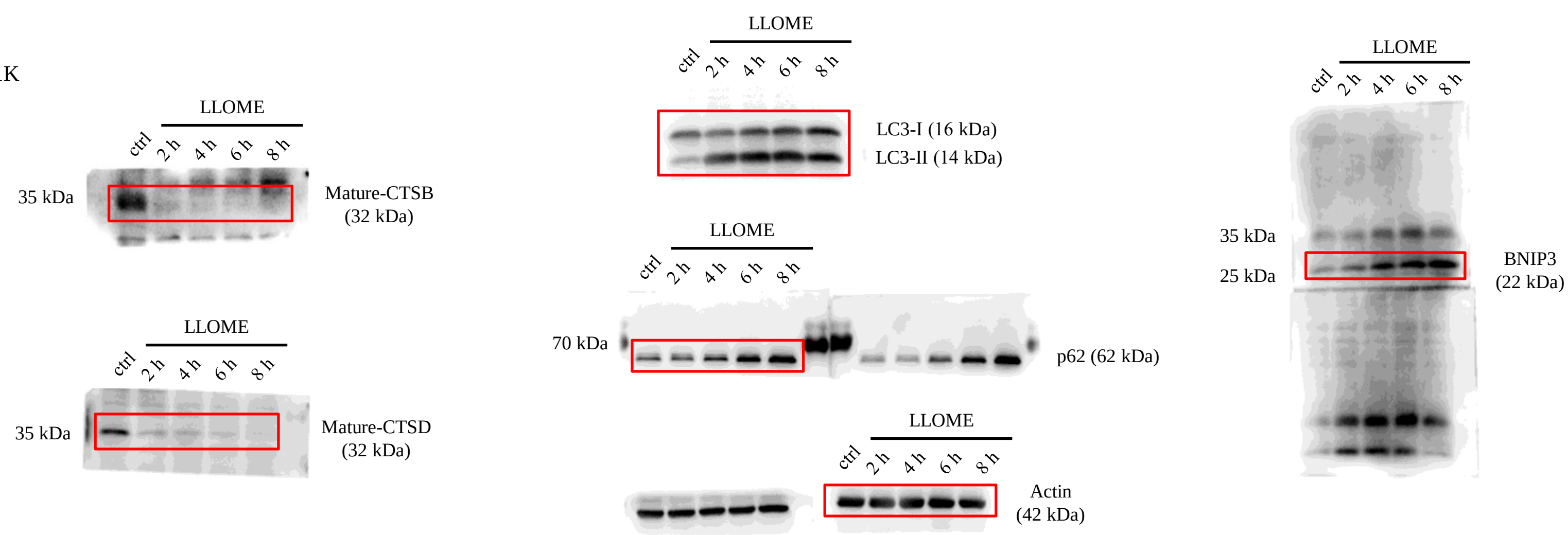

Figure. 2A

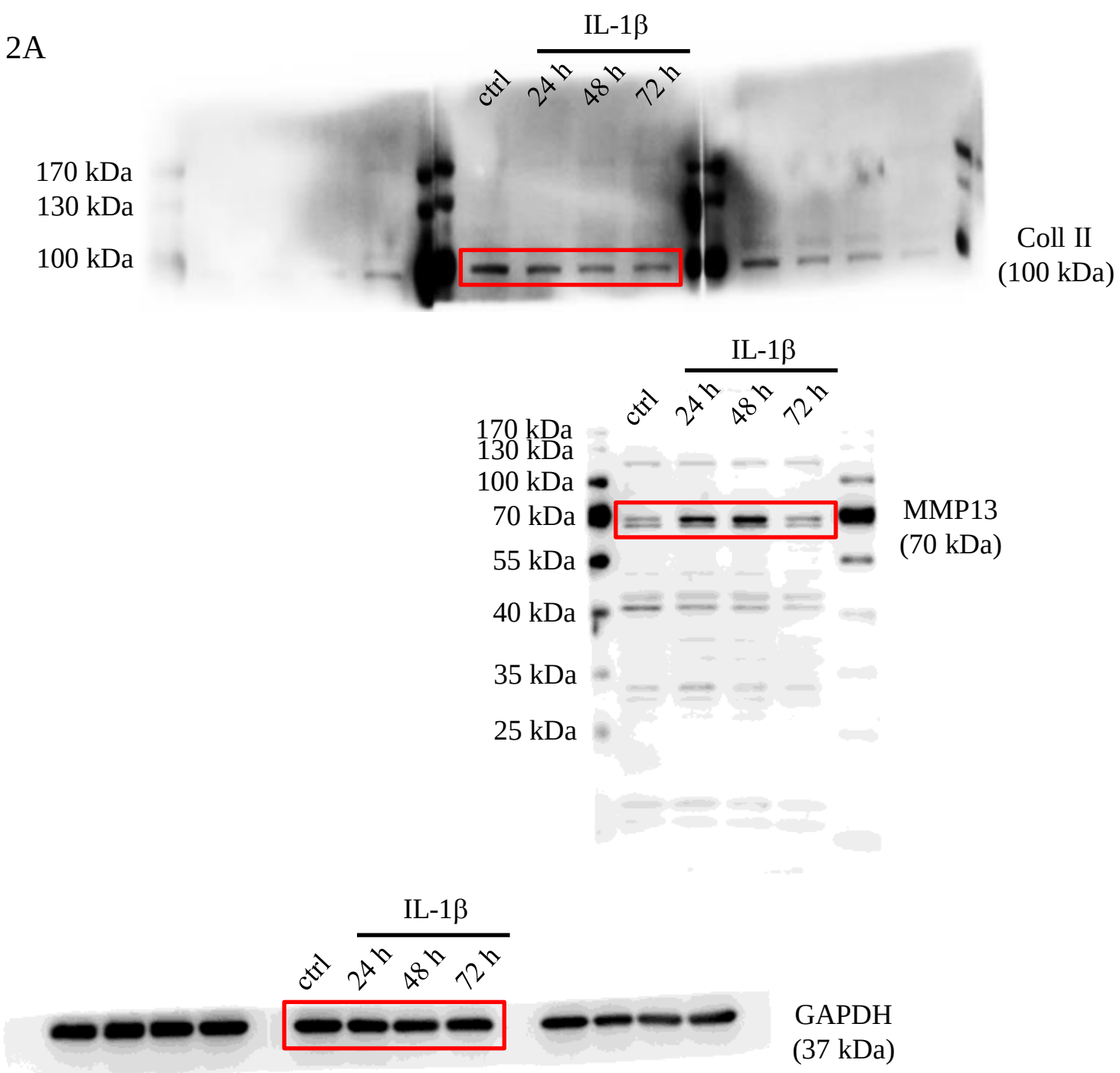

Figure. 2D

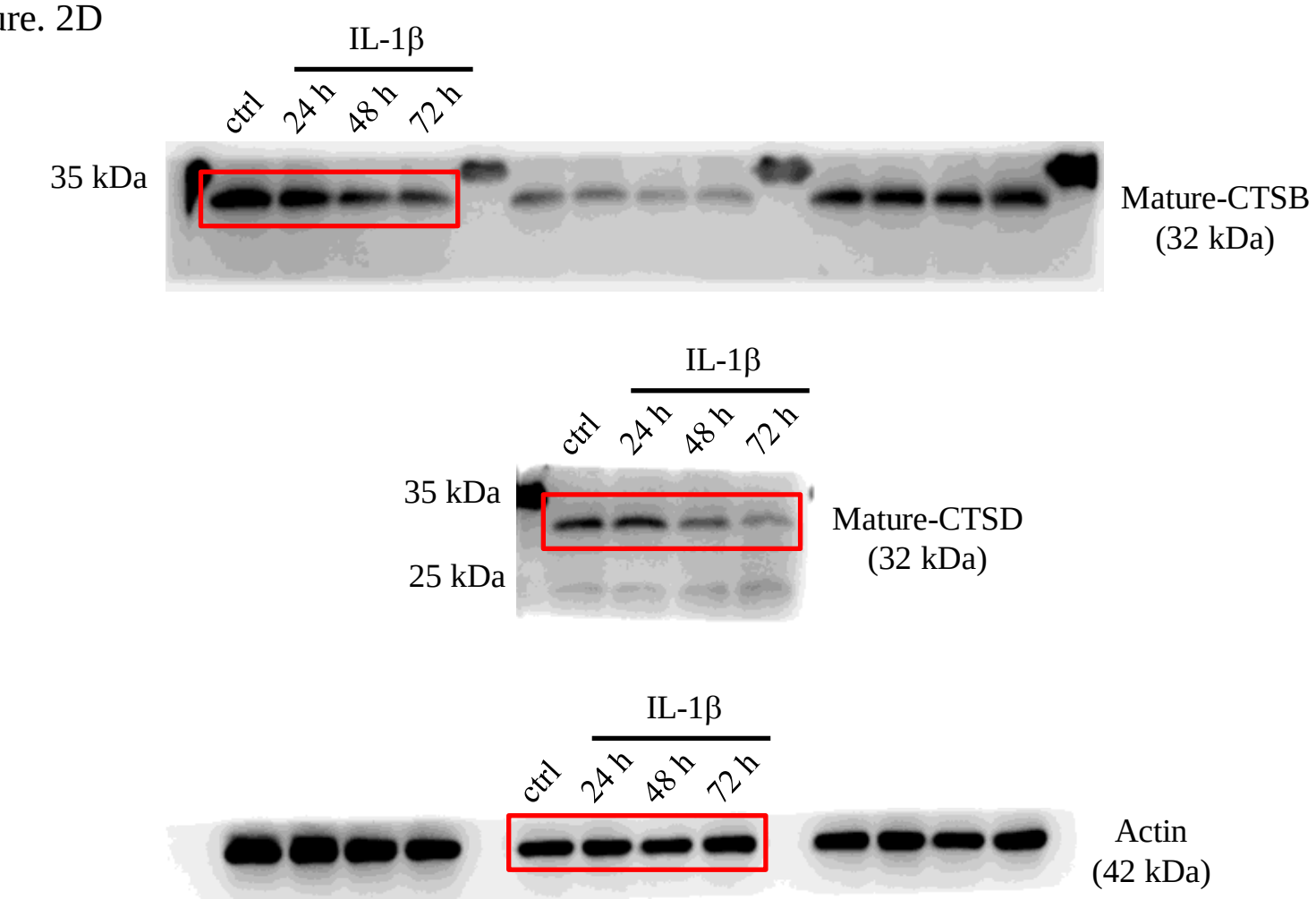

Figure. 2E

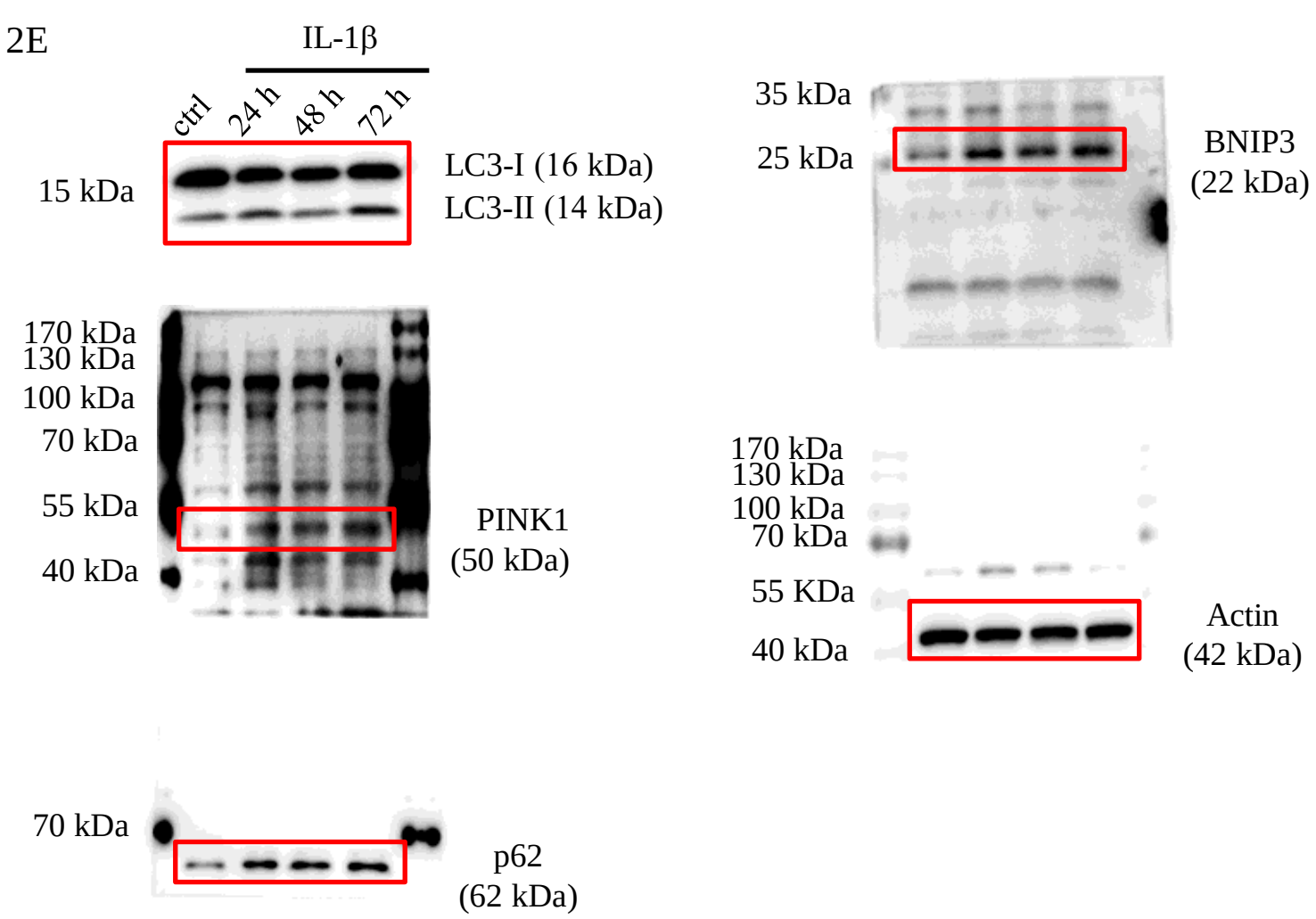

Figure. 2F

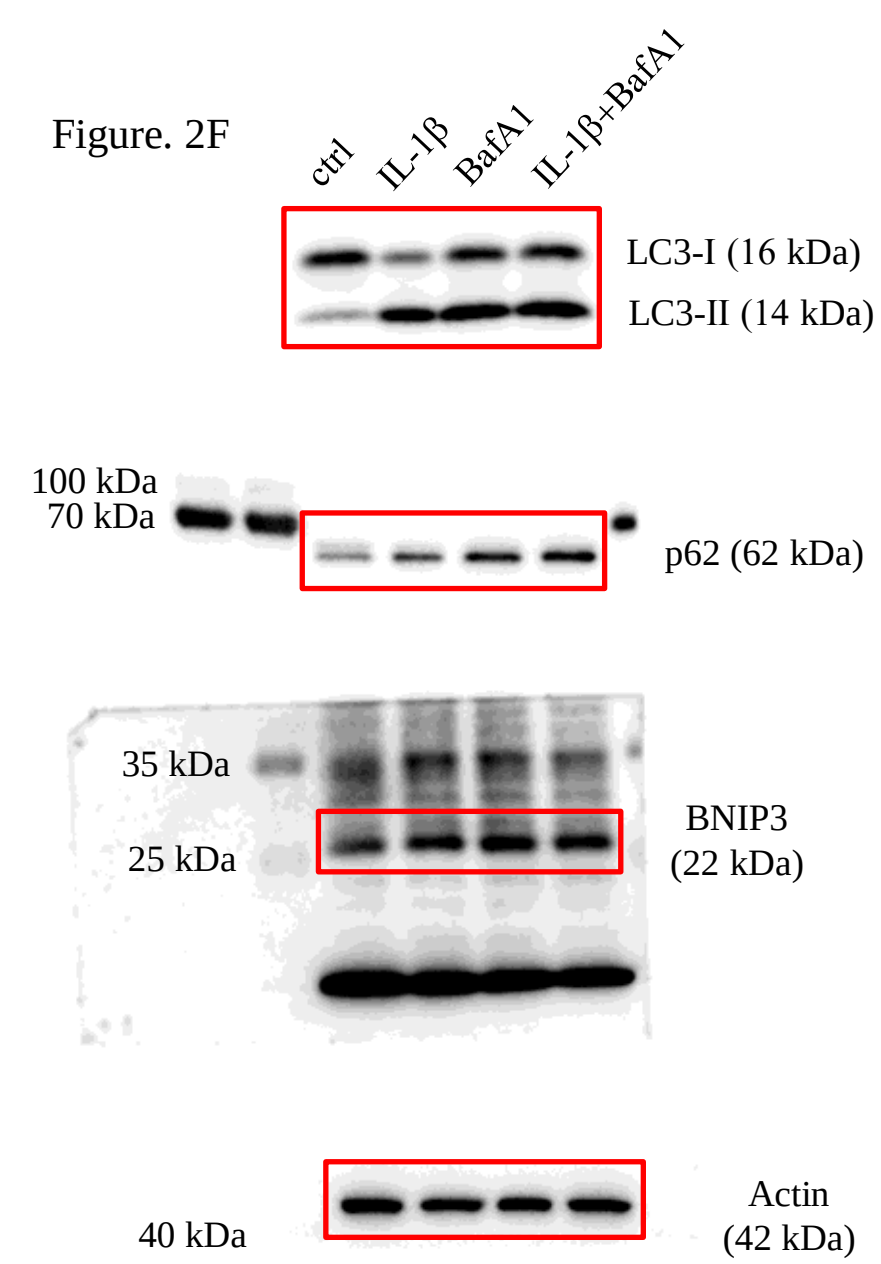

Figure. 3F

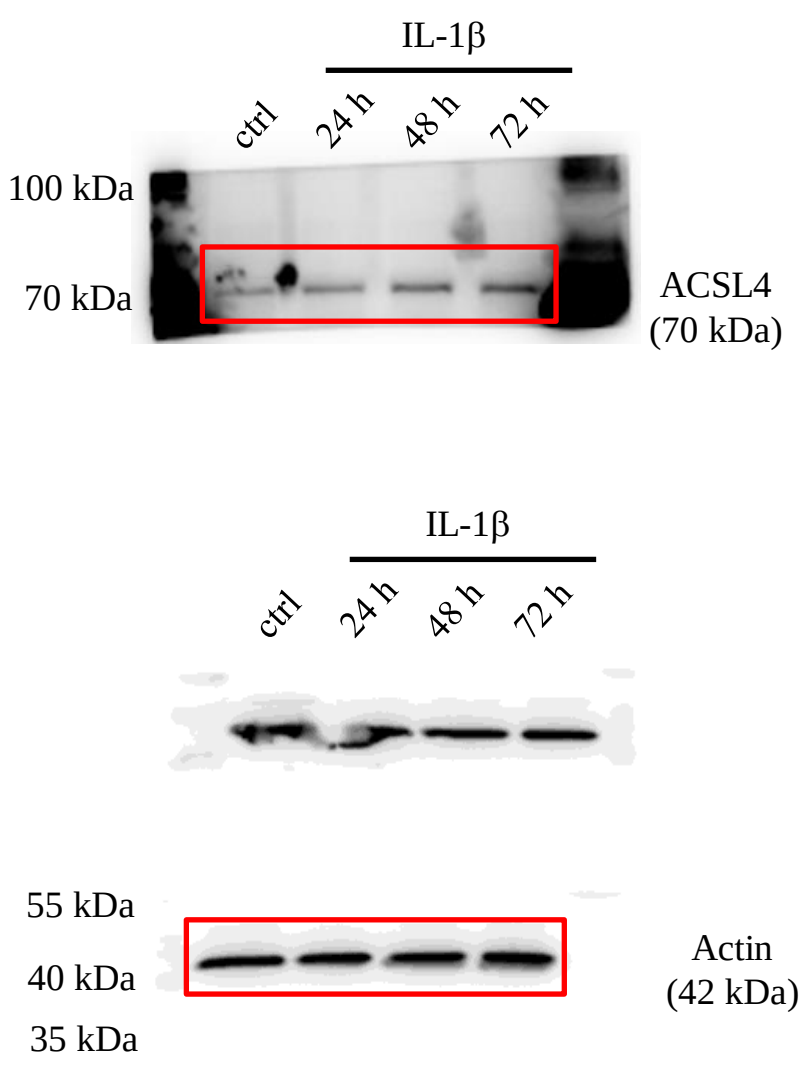

Figure. 3H

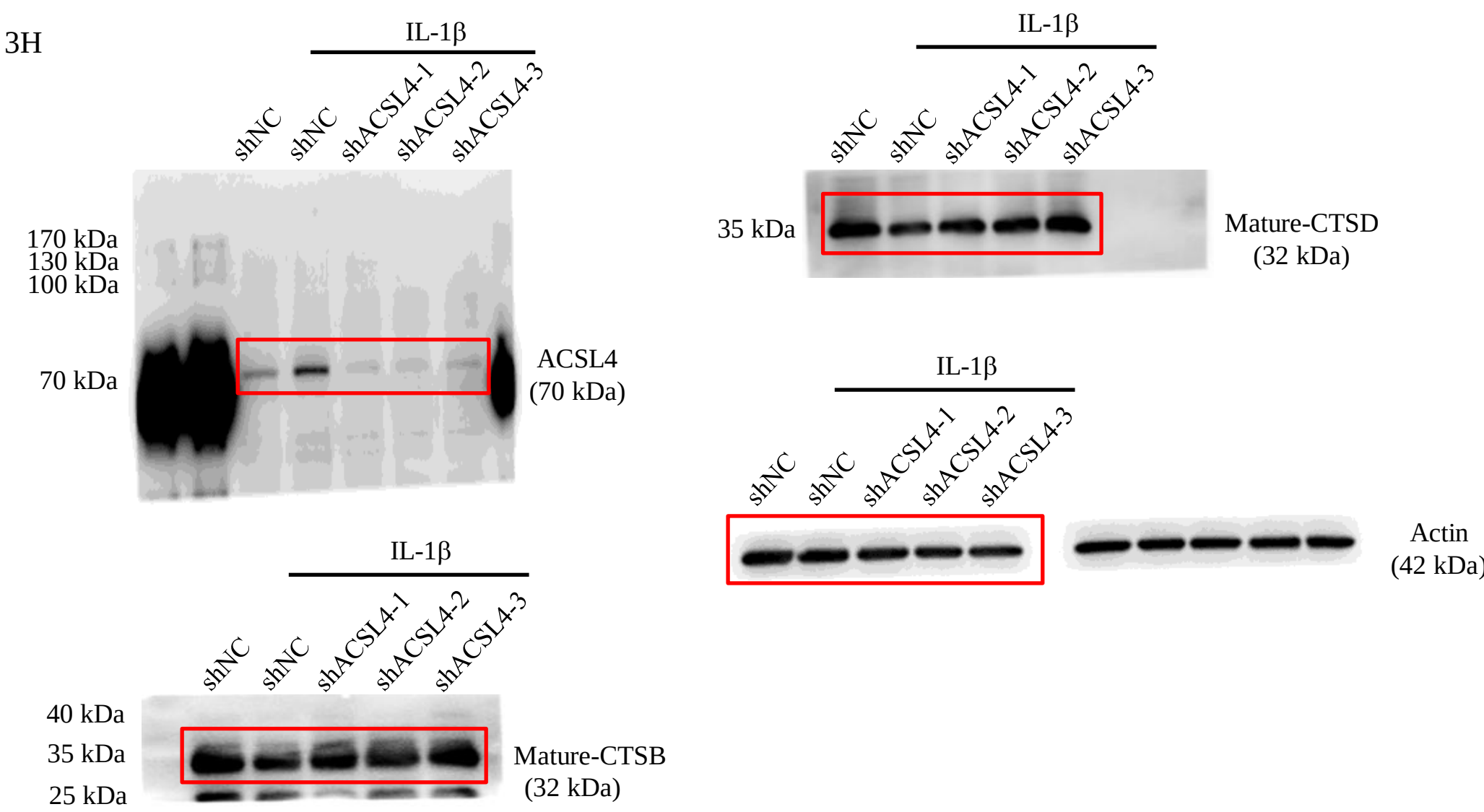

Figure. 3J

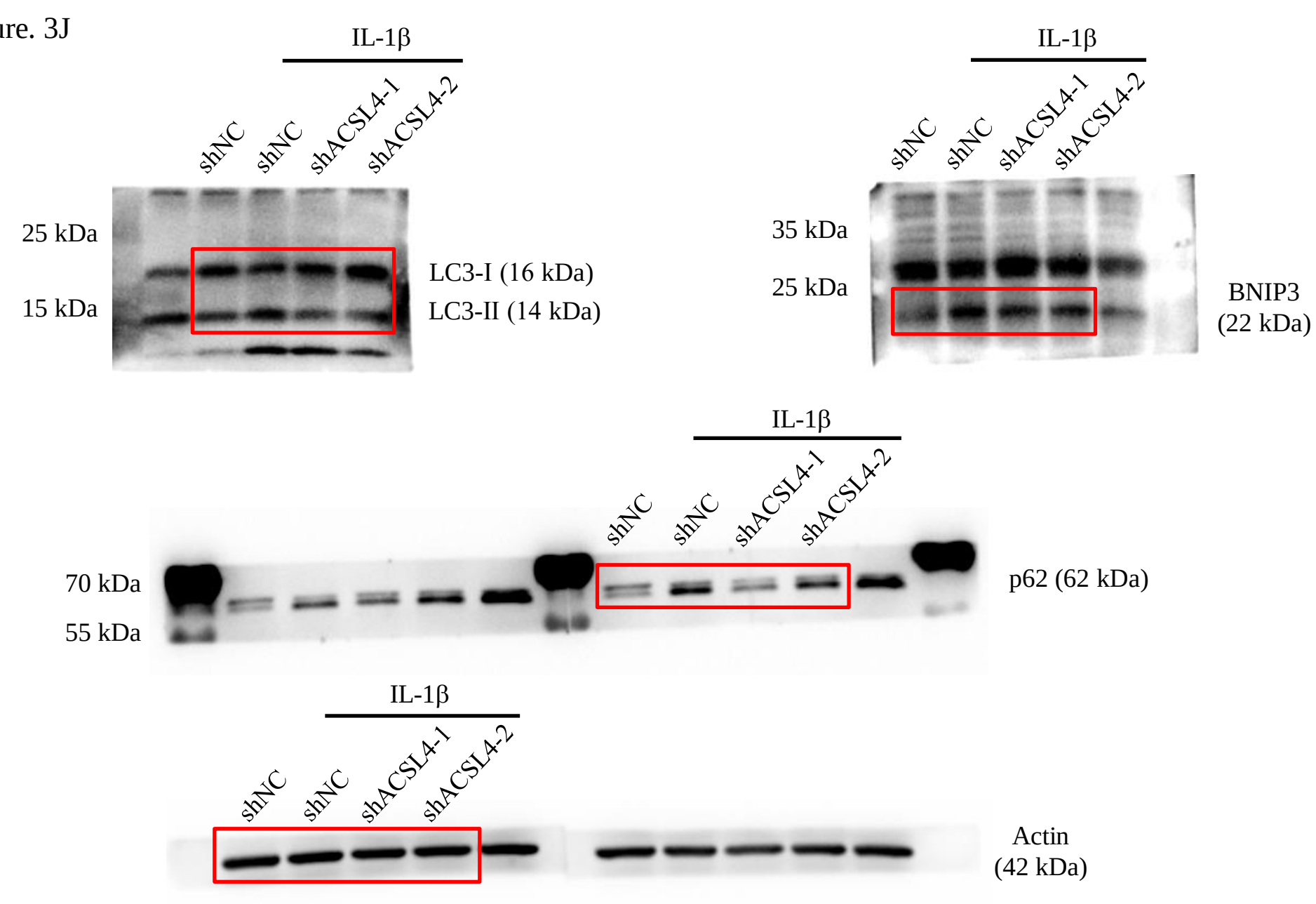

Figure. 4B

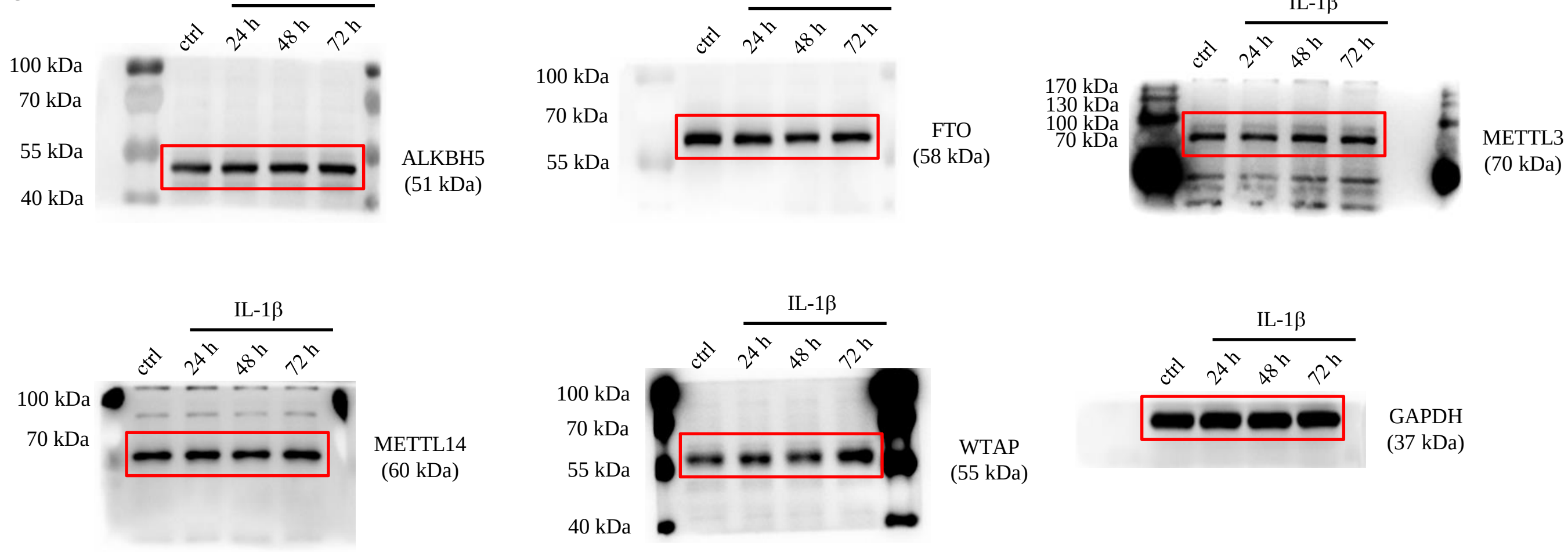

Figure. 4F

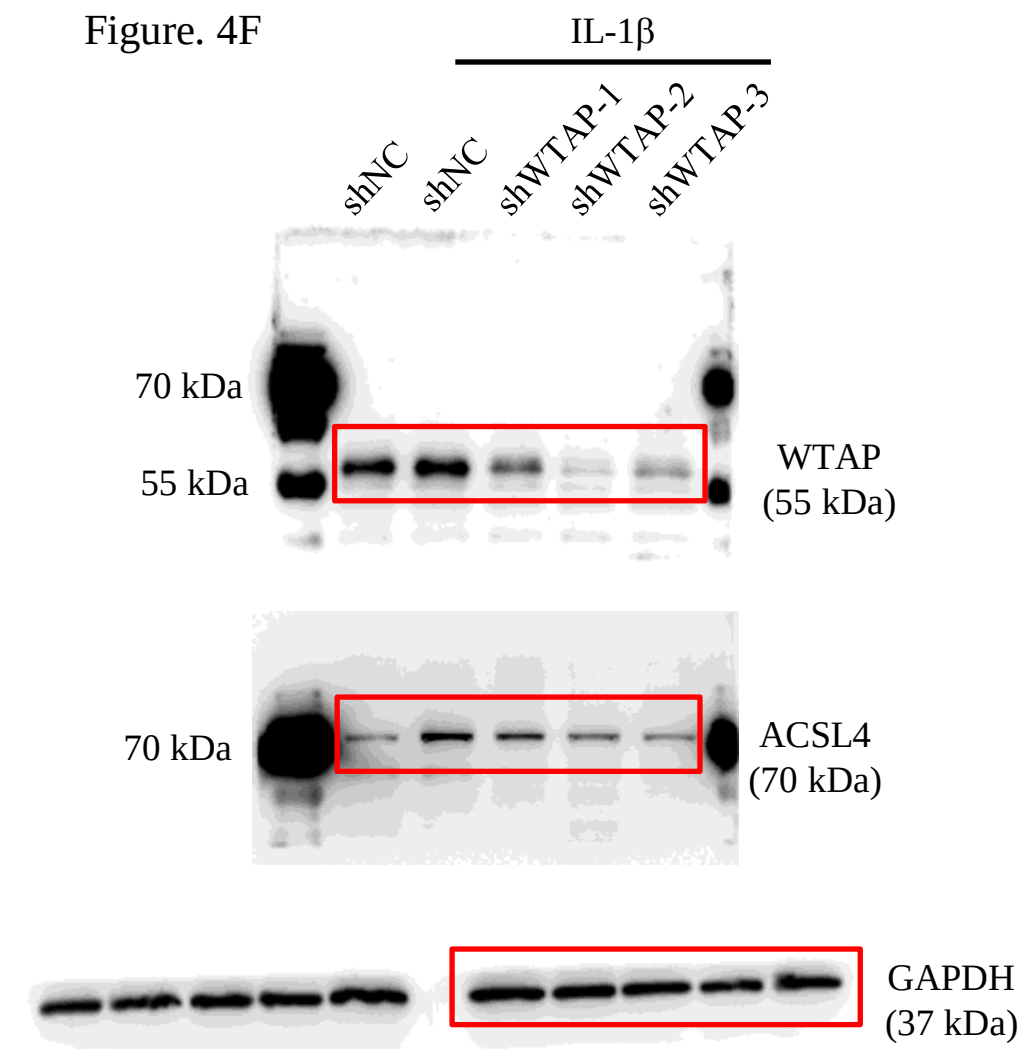

Figure. 4G

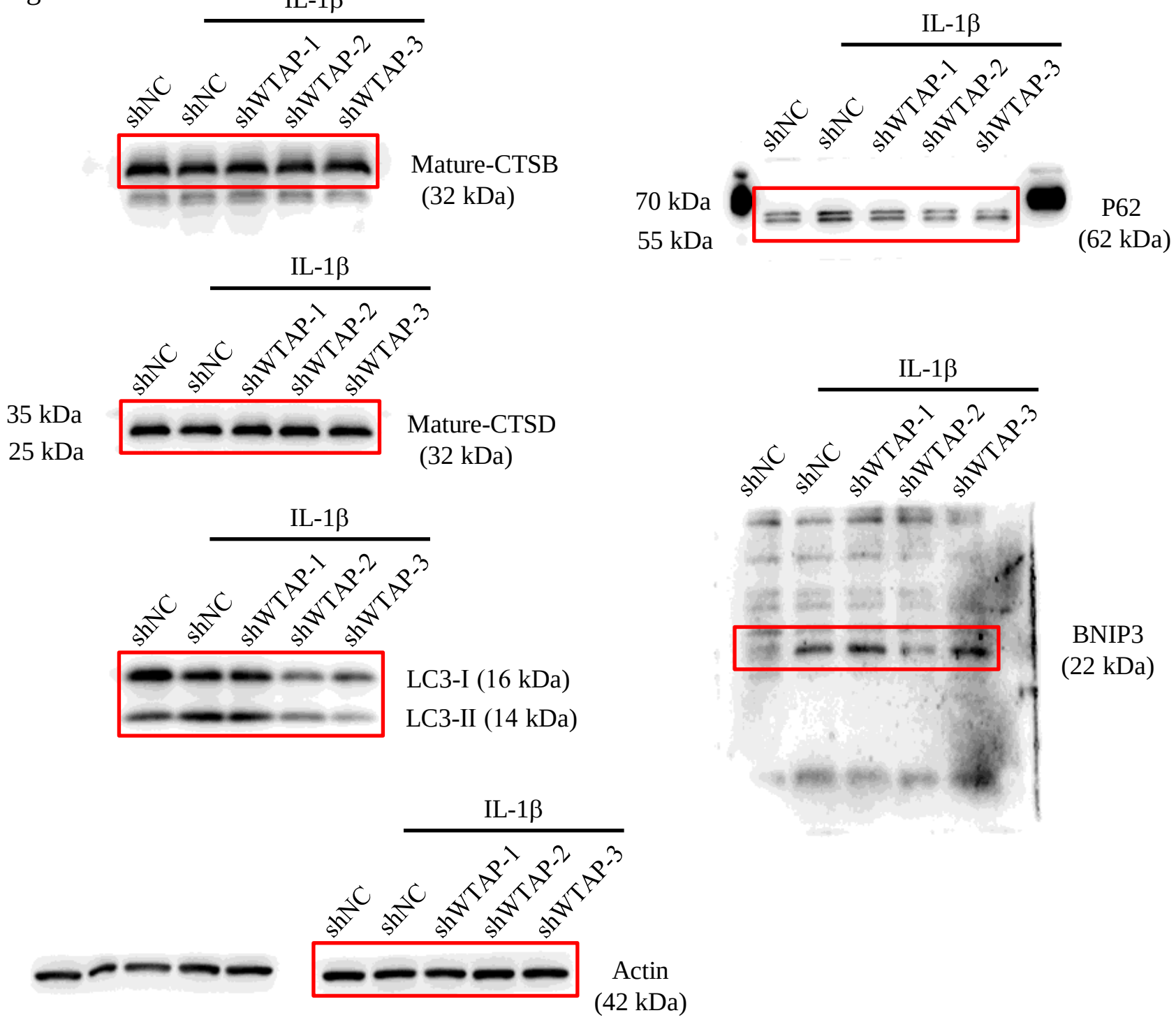

Figure. 4K

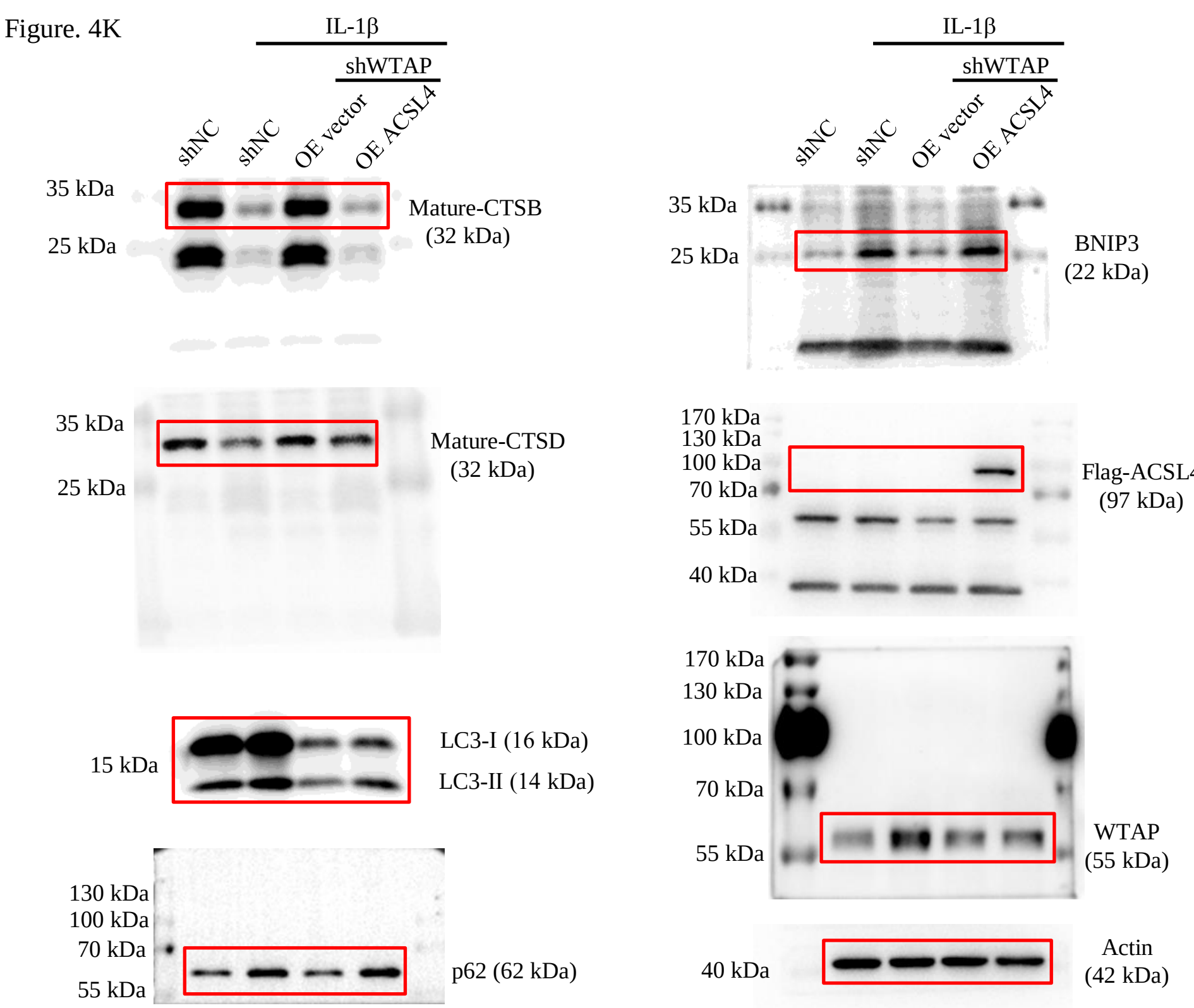

Figure. 5C

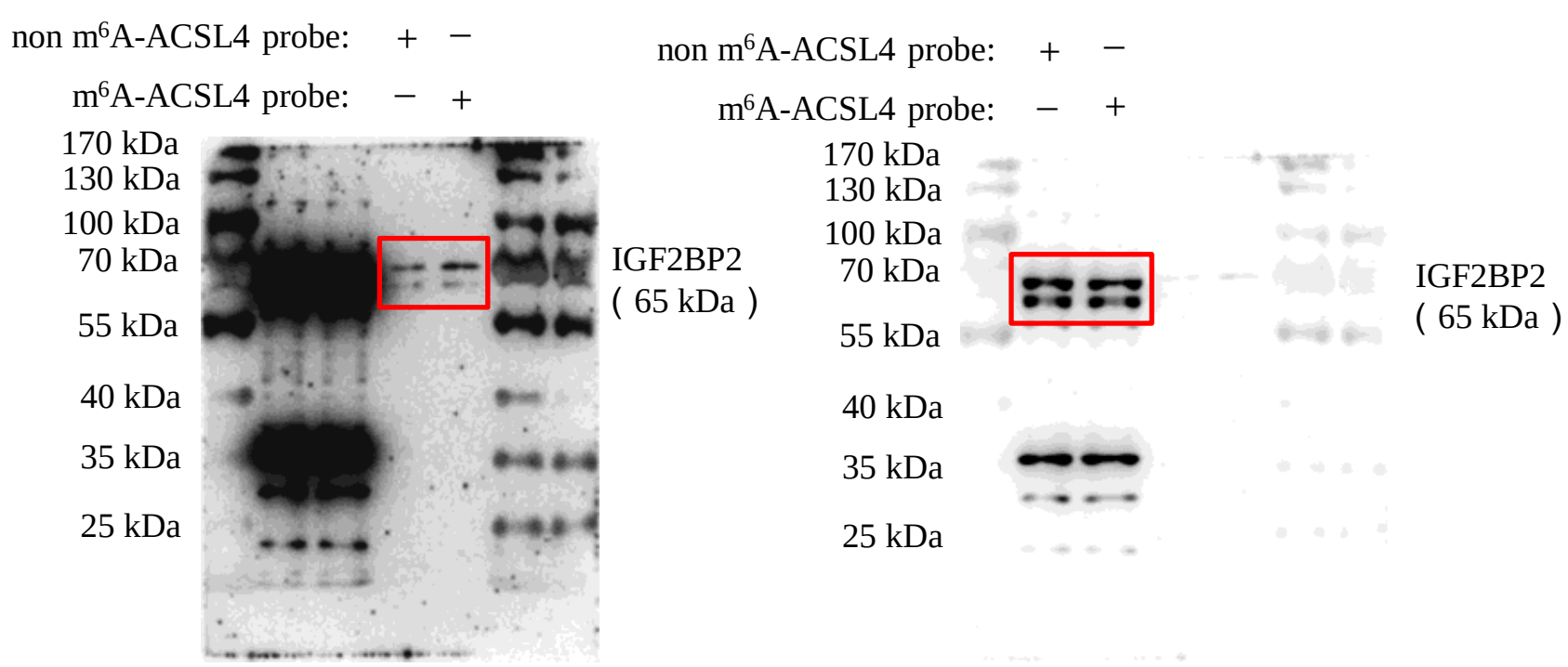

Figure. 5D

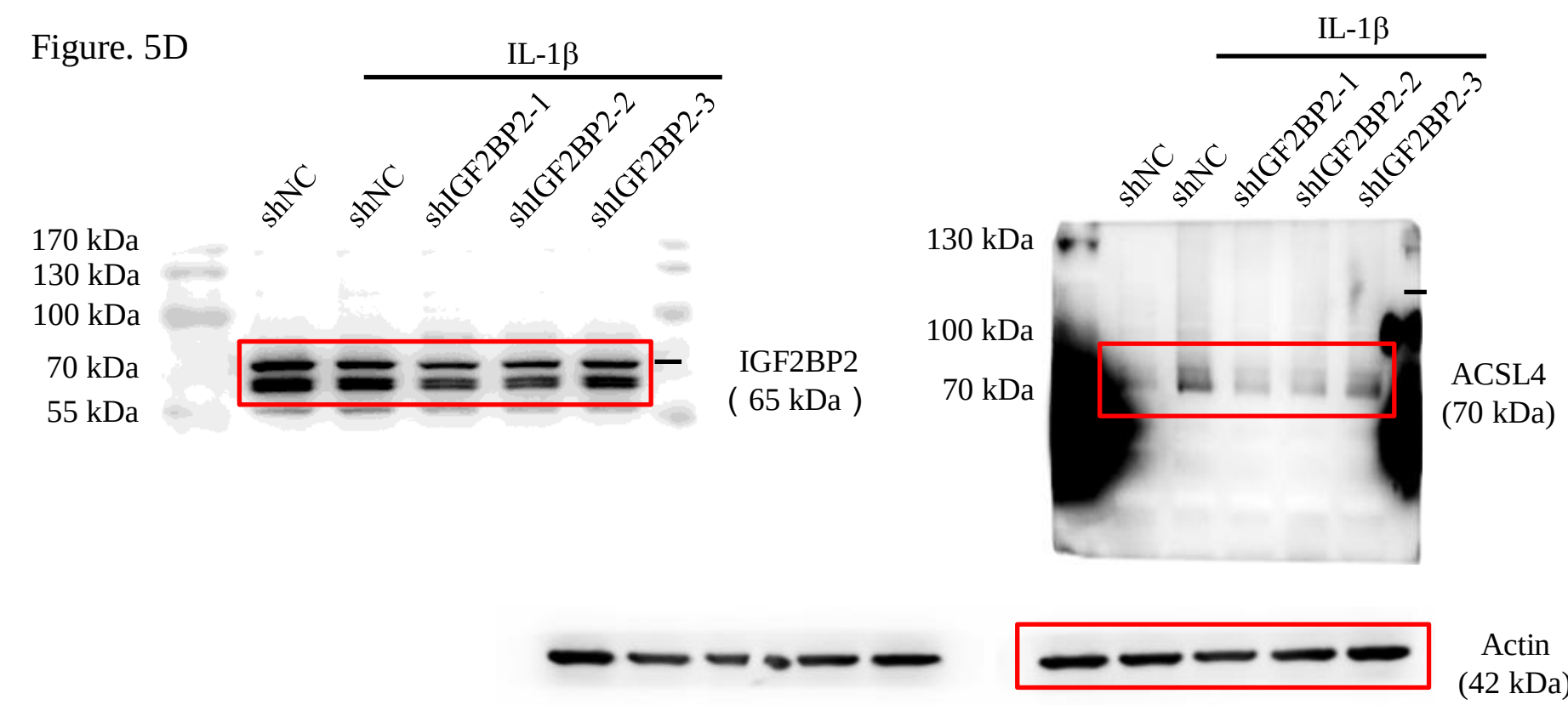

Figure. 5H

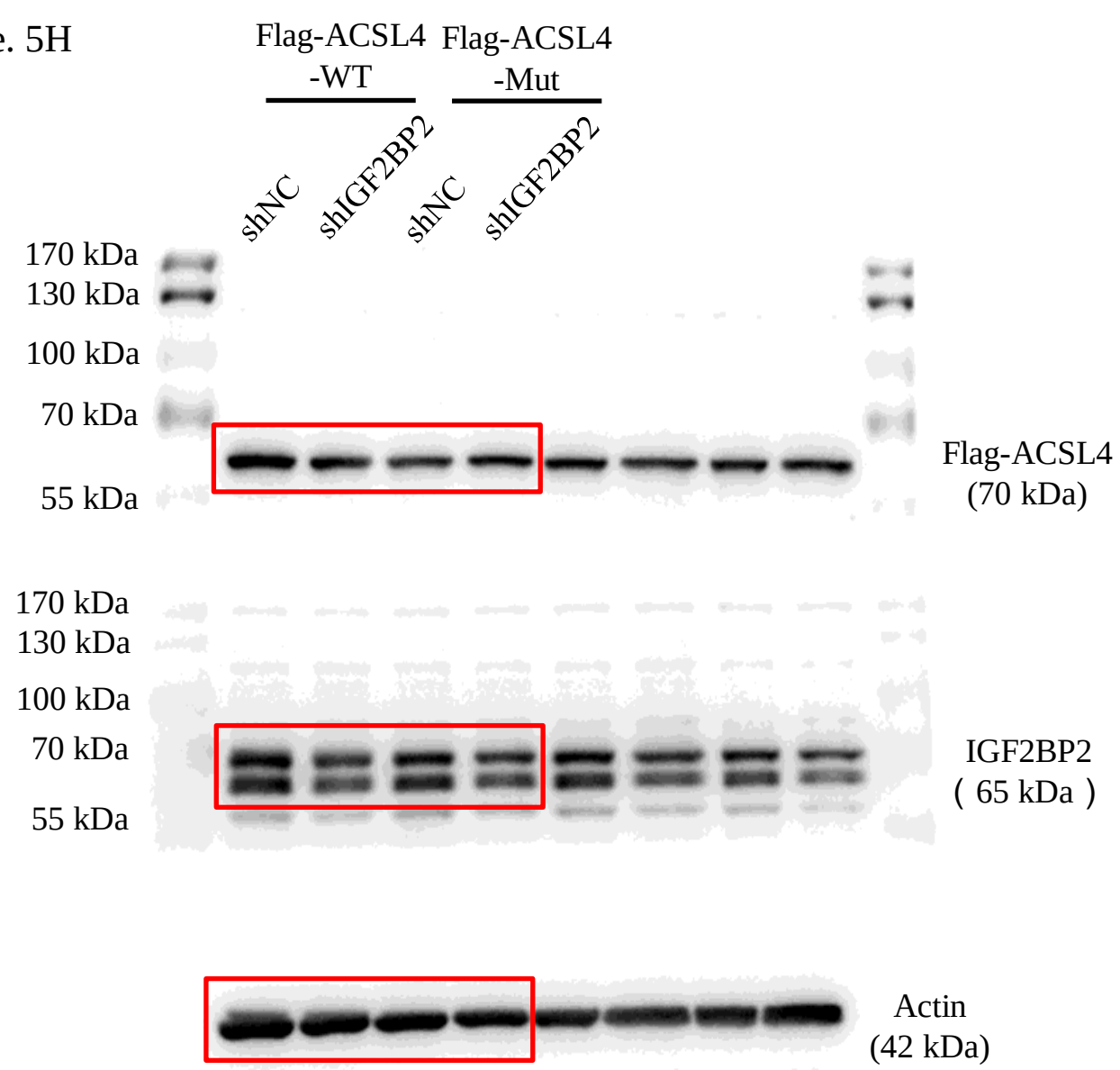

Figure. 5I

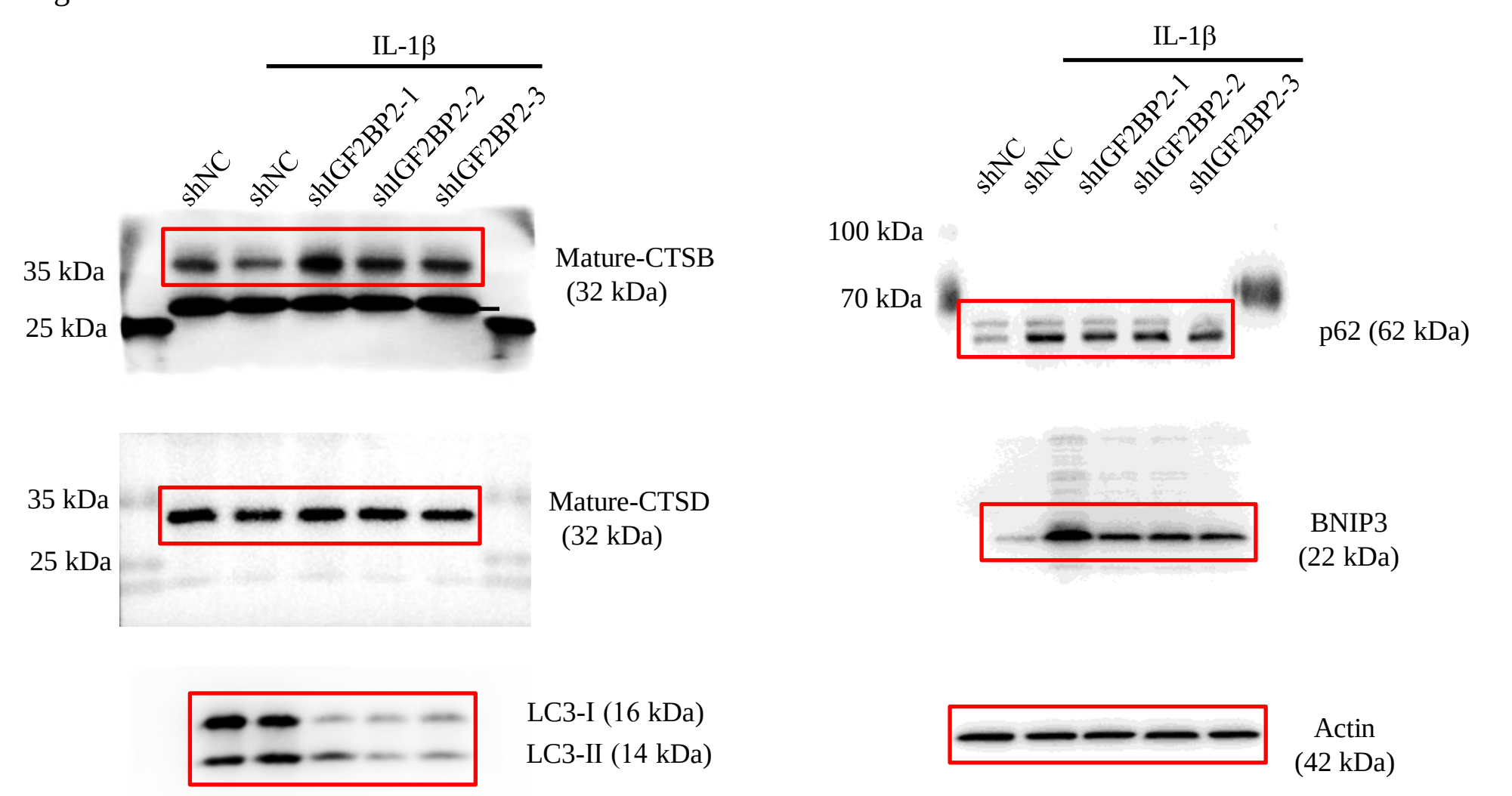

Figure. S2C

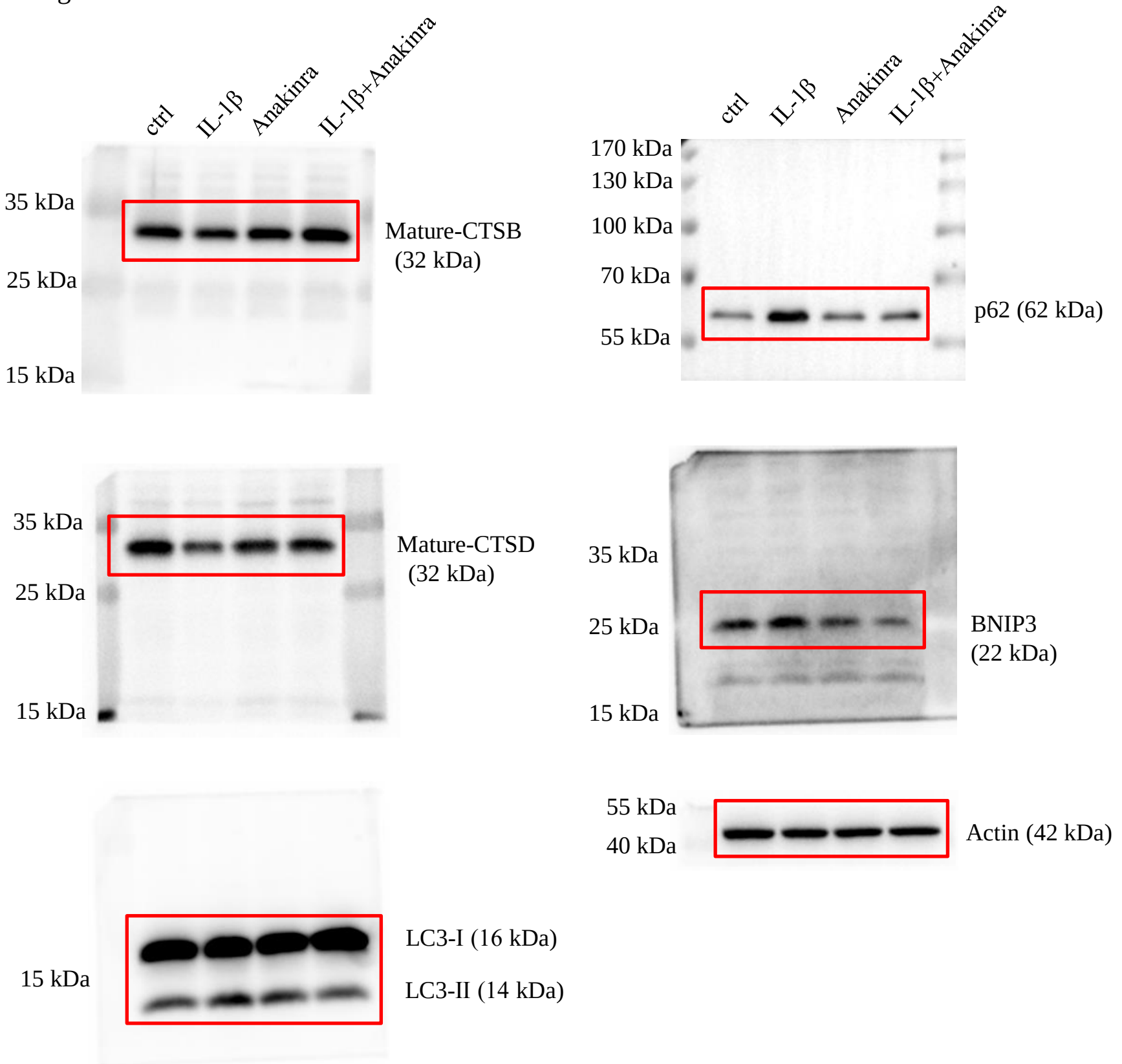

Figure. S7C

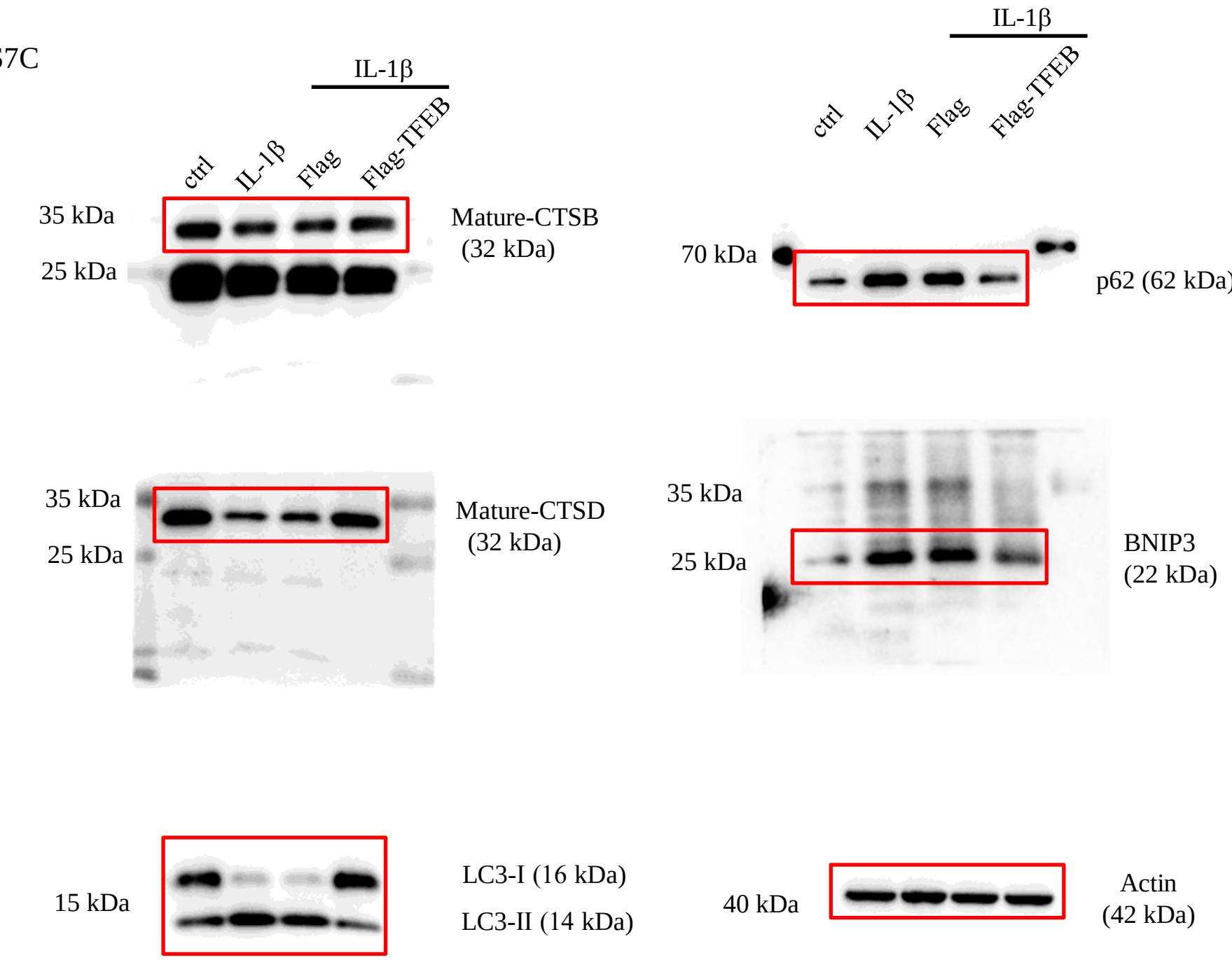

Supplement: Supplementary file 1 [file ADVS-9999-e77401-s002.pdf]
