# Supplementary material for: ACSL4‐Dependent Lysosomal Lipid Peroxidation Links WTAP‐Mediated m6A Modification to Intervertebral Disc Degeneration
Source: Adv Sci (Weinh). 2026 Aug 29:e77401. Online ahead of print. doi: 10.1002/advs.77401 (PMC13525423; doi:10.1002/advs.77401)
Supplement: Supplementary file 2 [file ADVS-9999-e77401-s001.docx]

Supplementary Materials for

ACSL4-dependent lysosomal lipid peroxidation links WTAP-mediated m6A modification to intervertebral disc degeneration

**This file includes:**

Supplementary Figure and Figure Legends

Supplementary Tables

**Supplementary Figure and Figure Legends**

**Figure S1.** ScRNA‑seq profiling of non‑degenerative and degenerative human NP tissues (GSE153066) uncovers cell cluster heterogeneity and impaired mitophagy‑lysosome signaling in degeneration. (A) UMAP visualization of human NP tissues identified nine different cell clusters. Each dot corresponds to a colored cell cluster. (B) UMAP plot depicting the proportions of cell types in control (CTL) and intervertebral disc degeneration (IDD) NP tissues. (C) Bar diagrams showing the grade distribution of each cell subset. (D-F) UMAP and violin plots showing the expressions of ACAN, COL2A1, and SOX9 in different cell clusters. (G, H) Dot plots showing KEGG pathways enriched among up-regulated (G) and down-regulated genes (H) in the NP cluster of IDD vs. CTL group. (I) GSEA enrichment curve of mitophagy pathway comparing IDD vs. CTL group. (J) GSEA enrichment curve of lysosome pathway comparing IDD vs. CTL group.

**Figure S2.** Anakinra rescues IL‑1β‑triggered impairment of mitophagic flux. (A) Representative IF images of IL-1β co-stained with Galectin-3 in mild- and severe-degenerated NP tissues, two-tailed unpaired *t*-test. (Scale bar = 100 μm) (B) Autophagic flux of the NP cells was assessed using the mCherry-GFP-LC3 reporter assay with or without the presence of IL-1β (20 ng/mL, 72 h) and Anakinra (400 ng/mL, 72 h). Yellow puncta (mCherry^+^/GFP^+^) represent autophagosomes and impaired degradation capacity of autolysosomes, two-way ANOVA with Tukey-Kramer test. (Scale bar = 10 μm, Enlarged scale bar = 2 μm) (C) Western blotting of mature-CTSB/CTSD and mitophagy marker proteins LC3-Ⅰ, LC3-Ⅱ, p62, and BNIP3 in NP cells with or without the presence of IL-1β (20 ng/mL, 72 h) and Anakinra (400 ng/mL, 72 h), two-way ANOVA with Tukey-Kramer test. Data are presented as mean ± SD. * vs ctrl, **p < 0.01 and ***p < 0.001; # vs IL-1β, ##p < 0.01 and ###p < 0.001.

**Figure S3.** GO terms and KEGG pathways of differential m6A peaks between control and IL-1β-treated NP cells. (A) Histogram displaying the enriched GO terms including biological process (BP), cellular component (CC) and molecular function (MF) of differential m6A peaks between control and IL-1β-treated NP cells. (B) Dot plot showing the enriched KEGG pathways of differential m6A peaks.

**Figure S4.** Quantitative analysis of WTAP, mature-CTSB, mature-CTSD, LC3-Ⅱ/Ⅰ, p62, and BNIP3 protein levels in Figure 4(K), two-way ANOVA with Tukey-Kramer test. Data are presented as mean ± SD. * vs shNC, ***p < 0.001; # vs shNC+IL-1β, ###p < 0.001; ∆ vs shWTAP+Vector+IL-1β, ^∆∆∆^p < 0.001.

**Figure S5.** IL‑1β increases the colocalization of ACSL4 with the lysosomal marker LAMP1 in NP cells. (A) Representative IF images of ACSL4 co-staining with LAMP1 in NP cells with or without the presence of IL-1β (20 ng/mL, 72 h). (Scale bar = 20 μm) (B) The colocalization of ACSL4 with LAMP1 was analyzed using Image J software, and the quantified data were plotted with GraphPad Prism 11.

**Figure S6.** Inhibition of the WTAP/ACSL4 axis attenuates IVDD by restoring lysosomal function and mitophagy flux. (A, B) Quantitative analysis of WTAP and ACSL4 protein levels in NP tissues from sham+AAV9-shNC, IVDD+AAV9-shNC, and IVDD+AAV9-shWTAP group, two-way ANOVA with Tukey-Kramer test. (C, D) Quantitative analysis of Coll Ⅱ and MMP13 protein levels in NP tissues from sham+AAV9-shNC, IVDD+AAV9-shNC, and IVDD+AAV9-shWTAP group, two-way ANOVA with Tukey-Kramer test. (E-J) Quantitative analysis of CTSB (E), CTSD (F), LC3 (G), p62 (H), BNIP3 (I) protein and 4-HNE (J) levels in NP tissues from sham+AAV9-shNC, IVDD+AAV9-shNC, and IVDD+AAV9-shWTAP group, two-way ANOVA with Tukey-Kramer test. Data are presented as mean ± SD. * vs sham+AAV9-shNC, *p < 0.05, **p < 0.01, ***p < 0.001; # vs IVDD+AAV9-shNC,#p < 0.05, ##p < 0.01, ###p < 0.001.

**Figure S7.** TFEB overexpression rescues IL-1β-suppressed lysosomal function and autophagic flux in NP cells. (A) Representative confocal microscopy images of NP cells co-transfected with mCherry-LGALS3 plasmids and either vector control or Flag-TFEB overexpression plasmids, followed by stimulation with IL-1β for 72 h, two-way ANOVA with Tukey-Kramer test. (Scale bar = 10 μm) (B) Autophagic flux of the NP cells treated as described in (A) was assessed using the mCherry-GFP-LC3 reporter assay. Yellow puncta (mCherry+/GFP+) represent autophagosomes and impaired degradation capacity of autolysosomes, two-way ANOVA with Tukey-Kramer test. (Scale bar = 10 μm, Enlarged scale bar = 1 μm) (C) Western blotting of mature-CTSB/CTSD and mitophagy marker proteins LC3-Ⅰ, LC3-Ⅱ, p62, and BNIP3 in NP cells treated as described in (A), two-way ANOVA with Tukey-Kramer test. Data are presented as mean ± SD. * vs ctrl, ***p < 0.001; # vs IL-1β+Flag, ###p < 0.001.

**Supplementary Tables**

**Table S1. Statistics table of m6A peaks**

| Sample/Group | Count | Average length | Median length | Min length | Max length |
| --- | --- | --- | --- | --- | --- |
| Con_1 | 23546 | 688.8 | 150 | 6 | 250179 |
| Con_2 | 26006 | 678.9 | 150 | 8 | 325106 |
| Con_3 | 24415 | 623.6 | 150 | 8 | 250179 |
| IL_1 | 23598 | 667.6 | 150 | 13 | 324931 |
| IL_2 | 20657 | 639.5 | 150 | 4 | 325106 |
| IL_3 | 22387 | 606.8 | 150 | 5 | 250179 |
| IL | 39651 | 856.2 | 175 | 4 | 417681 |
| Con | 41866 | 865.5 | 175 | 8 | 417731 |

**Table S2. Disc height index (DHI) of rat caudal intervertebral discs in each group**

| Group | Anterior heights of the intervertebral disc (a) | Middle heights of the intervertebral disc (b) | Posterior heights of the intervertebral disc (c) | Sagittal diameter of the upper vertebral bodies (A) | Sagittal diameter of the lower vertebral bodies (B) | Disc Height Index (DHI)  [(a + b + c)/3] / [(A + B)/2] |
| --- | --- | --- | --- | --- | --- | --- |

| Sham+AAV9-shNC | 14.727 | 13.537 | 13.597 | 64 | 65.347 | 0.0539388 |
| --- | --- | --- | --- | --- | --- | --- |
|  | 12 | 10.667 | 14.727 | 72.012 | 72.012 | 0.0432728 |
|  | 14.667 | 13.333 | 12 | 69.538 | 73.382 | 0.0466461 |
|  | 13.333 | 10.667 | 14.727 | 76.292 | 77.379 | 0.0420020 |
|  | 16.055 | 14.667 | 14.667 | 69.385 | 76.012 | 0.0520288 |
| IVDD+AAV9-shNC | 10.727 | 10.667 | 10.074 | 58.088 | 70.98 | 0.040635 |
|  | 9.428 | 10.75 | 8 | 74.774 | 82.677 | 0.029827 |
|  | 9.333 | 10.75 | 8 | 70.717 | 74.857 | 0.032152 |
|  | 8 | 8 | 9.428 | 76.047 | 72.111 | 0.037497 |
|  | 9.707 | 10.75 | 8.11 | 56.395 | 70.868 | 0.037412 |
| IVDD+AAV9-shWTAP | 13.4 | 12.074 | 14.727 | 68.013 | 66.68 | 0.049744 |
|  | 13.333 | 12.293 | 10.75 | 68.013 | 70.717 | 0.043701 |
|  | 12.074 | 12.995 | 12.75 | 70.746 | 70.187 | 0.044725 |
|  | 13.4 | 12.074 | 13.333 | 73.345 | 72.111 | 0.044466 |
|  | 12.074 | 13.4 | 14.907 | 72.049 | 73.333 | 0.046293 |

**Table S3. Histological grading score of rat caudal intervertebral discs in each group**

| Group | AF morphology | Border between AF and NP | Cellularity of the NP | Matrix of the NP | Histological grading score |
| --- | --- | --- | --- | --- | --- |
| Sham+AAV9-shNC | 1 | 1 | 1 | 1 | 4 |
|  | 1 | 2 | 1 | 1 | 5 |
|  | 2 | 1 | 1 | 1 | 5 |
|  | 1 | 1 | 1 | 1 | 4 |
|  | 1 | 1 | 1 | 1 | 4 |
| IVDD+AAV9-shNC | 3 | 3 | 3 | 3 | 12 |
|  | 3 | 3 | 3 | 3 | 12 |
|  | 2 | 3 | 3 | 3 | 11 |
|  | 3 | 3 | 3 | 3 | 12 |
|  | 2 | 3 | 3 | 3 | 11 |
| IVDD+AAV9-shWTAP | 2 | 1 | 2 | 2 | 7 |
|  | 1 | 1 | 2 | 2 | 6 |
|  | 2 | 1 | 2 | 2 | 7 |
|  | 2 | 2 | 2 | 2 | 8 |
|  | 2 | 2 | 2 | 2 | 8 |

**Table S4. Patient’s information**

| No. | Sex | Age | Diagnosis | Surgical  Level | Pfirrmann  score |
| --- | --- | --- | --- | --- | --- |
| 1 | M | 35 | Lumbar disc herniation | L4/L5 | II |
| 2 | M | 18 | Lumbar disc herniation | L4/L5 | II |
| 3 | M | 28 | Lumbar disc herniation | L5/S1 | II |
| 4 | F | 44 | Lumbar disc herniation | L4/L5 | II |
| 5 | M | 41 | Lumbar disc herniation | L5/S1 | II |
| 6 | M | 35 | Lumbar disc herniation | L5/S1 | IV |
| 7 | F | 18 | Lumbar disc herniation | L4/L5 | IV |
| 8 | M | 23 | Lumbar disc herniation | L5/S1 | IV |
| 9 | M | 25 | Lumbar disc herniation | L5/S1 | IV |
| 10 | M | 33 | Lumbar disc herniation | L5/S1 | IV |

**Table S5. shRNA sequences**

| shRNA | Target sequence (5’ to 3’) | |
| --- | --- | --- |
| sh-WTAP | sh-WTAP-1 | GTTATGGCAAGAGATGAGTTA |
|  | sh-WTAP-2 | GCAAGAGTGTACTACTCAAAT |
|  | sh-WTAP-3 | GGCAAGTACACAGATCTTAAC |
| sh-ACSL4 | sh-ACSL4-1 | GCAGTAGTTCATGGGCTAAAT |
|  | sh-ACSL4-2 | GCATCATAGCAATTTGATAGC |
|  | sh-ACSL4-3 | GCTGCAAATGCCATGAAATTG |
| sh-IGF2BP2 | sh-IGF2BP2-1 | CTTAACCAGTGCAGAAGTCAT |
|  | sh-IGF2BP2-2 | CGGATCTTTGGGAAACTGAAA |
|  | sh-IGF2BP2-3 | CAGTGCTGAGATAGAGATTAT |

**Table S6. Primer sequences for RT-qPCR and MeRIP-qPCR**

| Gene | Primer sequence (5’ to 3’) | |
| --- | --- | --- |
| ACSL4 primers for MeRIP-qPCR | Forward | GAGCCATGGACCCCTGAAACT |
|  | Reverse | GCTACCTCCTGCACAACTGTC |
| ACSL4 primers for RT-qPCR | Forward | GGCAGAGTTGGAGCACCTCTTA |
|  | Reverse | TCACCTCTGGGGTTTGGCTTG |
| GAPDH primers for RT-qPCR | Forward | GCACCGTCAAGGCTGAGAAC |
|  | Reverse | TGGTGAAGACGCCAGTGGA |

**Table S7. RNA oligo sequences**

| RNA oligo | m6A-ACSL4 probe | Non-m6A-ACSL4 probe |
| --- | --- | --- |
| Sequence  (5’ to 3’) | ACUGAUGCUUUCAA/6-MerA/CUGAAAAGGAAGGAGCUGAGGAACCAUUACCUCAAAG/6-MerA/CAUUGAACGAAUGUAUGGGGGCAAAUAAAAUGUUGUUGUCUUAUUGA | ACUGAUGCUUUCAAACUGAAAAGGAAGGAGCUGAGGAACCAUUACCUCAAAGACAUUGAACGAAUGUAUGGGGGCAAAUAAAAUGUUGUUGUCUUAUUGA |

**Table S8. Details of key resources**

| Reagent or resource | Cat. number | Applications | Source |
| --- | --- | --- | --- |
| Antibodies |  |  |  |
| m⁶A antibody | 202003 | MeRIP | Synaptic Systems |
| Rabbit anti-CTSB | 12216-1-AP | WB: 1:1000  IF: 1:100 | Proteintech |
| Rabbit anti-CTSD | 21327-1-AP | WB: 1:1000  IF: 1:100 | Proteintech |
| Rabbit anti-WTAP | 56501S | WB: 1:1000  IF: 1:100 | Cell Signaling Technology |
| Rabbit anti-ACSL4 | ab155282 | WB: 1:1000  IF: 1:100 | Abcam |
| Rabbit anti-LC3A/B | 12741 | WB: 1:1000  IF: 1:100 | Cell Signaling Technology |
| Rabbit anti-p62 | 23214 | IF: 1:100  For rat | Cell Signaling Technology |
| Rabbit anti-p62 | 7695 | IF: 1:100  For human | Cell Signaling Technology |
| Rabbit anti-p62 | 39749 | WB: 1:1000 | Cell Signaling Technology |
| Mouse anti-LAMP1 | 21997-1-AP | IF: 1:100 | Proteintech |
| Mouse anti-BNIP3 | sc-56167 | WB: 1:500  IF: 1:100 | Santa Cruz Biotechnology |
| Rabbit anti-4-HNE | HY-P81208 | IF: 1:200 | MedChemExpress |
| Rabbit anti-PINK1 | 6946 | WB: 1:1000 | Cell Signaling Technology |
| Rabbit anti-ALKBH5 | 16837-1-AP | WB: 1:1000 | Proteintech |
| Rabbit anti-FTO | A3861 | WB: 1:1000 | ABclonal |
| Rabbit anti-METTL14 | A8530 | WB: 1:1000 | ABclonal |
| Rabbit anti-METTL3 | A8370 | WB: 1:1000 | ABclonal |
| Rabbit anti-IGF2BP2 | 11601-1-AP | WB: 1:1000 | Proteintech |
| Rabbit anti-Actin | AC026 | WB: 1:5000 | ABclonal |
| Rabbit anti-Flag | AE004 | WB: 1:2000 | ABclonal |
| HRP-linked goat anti-Rabbit antibody | GB23303 | IF: 1:300 | Servicebio |
| Goat anti-Mouse IgG, Alexa Fluor™ 488 | A-11001 | IF: 1:500 | Invitrogen |
| Goat anti-Mouse IgG, Alexa Fluor™ 594 | A-11005 | IF: 1:500 | Invitrogen |
| Goat anti-Mouse IgG, DyLight™ 650 | 84545 | IF: 1:500 | Invitrogen |
| Goat Anti-Rabbit IgG, Alexa Fluor™ 488 | A-11008 | IF: 1:500 | Invitrogen |
| Goat Anti-Rabbit IgG, Alexa Fluor™ 594 | A-11012 | IF: 1:500 | Invitrogen |
| Goat Anti-Rabbit IgG, DyLight™ 650 | 84546 | IF: 1:500 | Invitrogen |
| Reagents |  |  |  |
| Lipofectamine 3000 | L3000015 |  | Thermo Fisher Scientific |
| TRIzol | 15596026 |  | Thermo Fisher Scientific |
| actinomycin D | HY-17559 |  | MedChemExpress |
| IL-1β | HY-P7028 |  | MedChemExpress |
| BODIPY581/591 C11 | D3861 |  | Thermo Fisher Scientific |
| MitoBright LT Deep Red | MT12 |  | DOJINDO |
| Rhodamine 123 | C2007 |  | Beyotime |
| MitoSox Red | 40778ES50 |  | Yeasen |
| 3, 3’-diaminobenzidine (DAB) | HY-W014212 |  | MedChemExpress |
| Critical Commercial Assays |  |  |  |
| Dynabeads mRNA DIRECT Purification Kit | 61011 |  | Thermo Fisher Scientific |
| Magnesium RNA Fragmentation Module | E6150 |  | New England Biolabs |
| NEBNext Ultra II RNA Library Prep Kit | E7770 |  | New England Biolabs |
| EpiQuik CUT&RUN m^6^A RNA Enrichment Kit | P-9018-24 |  | EpigenTek |
| Pierce Magnetic RNA-Protein Pull-Down Kit | 20164 |  | Thermo Fisher Scientific |
| Lipid Peroxidation (MDA) Assay Kit | HY-K0319 |  | MedChemExpress |
| Hematoxylin-Eosin(HE) Stain Kit | G1120 |  | Solarbio |
| Alcian Blue Cartilage Stain Kit | G2542 |  | Solarbio |
| Modified Saffron-O And Fast Green Stain Kit | G1371 |  | Solarbio |
